# Individualized interactomes for network-based precision medicine in hypertrophic cardiomyopathy with implications for other clinical pathophenotypes

**DOI:** 10.1038/s41467-021-21146-y

**Published:** 2021-02-08

**Authors:** Bradley A. Maron, Rui-Sheng Wang, Sergei Shevtsov, Stavros G. Drakos, Elena Arons, Omar Wever-Pinzon, Gordon S. Huggins, Andriy O. Samokhin, William M. Oldham, Yasmine Aguib, Magdi H. Yacoub, Ethan J. Rowin, Barry J. Maron, Martin S. Maron, Joseph Loscalzo

**Affiliations:** 1 Division of Cardiovascular Medicine, Department of Medicine, Brigham and Women’s Hospital and Harvard Medical School, Boston, MA USA; 2 Channing Division of Network Medicine, Department of Medicine, Brigham and Women’s Hospital and Harvard Medical School, Boston, MA USA; 3 Division of Cardiovascular Medicine, University of Utah School of Medicine, Salt Lake City, UT USA; 4 Nora Eccles Harrison Cardiovascular Research and Training Institute (CVRTI), University of Utah School of Medicine, Salt Lake City, UT USA; 5 Hypertrophic Cardiomyopathy Center, Cardiology Division, Tufts Medical Center, Boston, MA USA; 6 Division of Pulmonary and Critical Care Medicine, Department of Medicine, Brigham and Women’s Hospital and Harvard Medical School, Boston, MA USA; 7 Department of Cardiac Surgery, Imperial College of London, London, UK; 8 The Magdi Yacoub Heart Center, Aswan, Egypt

**Keywords:** Network topology, Cardiac hypertrophy, Molecular medicine

## Abstract

Progress in precision medicine is limited by insufficient knowledge of transcriptomic or proteomic features in involved tissues that define pathobiological differences between patients. Here, myectomy tissue from patients with obstructive hypertrophic cardiomyopathy and heart failure is analyzed using RNA-Seq, and the results are used to develop individualized protein-protein interaction networks. From this approach, hypertrophic cardiomyopathy is distinguished from dilated cardiomyopathy based on the protein-protein interaction network pattern. Within the hypertrophic cardiomyopathy cohort, the patient-specific networks are variable in complexity, and enriched for 30 endophenotypes. The cardiac Janus kinase 2-Signal Transducer and Activator of Transcription 3-collagen 4A2 (JAK2-STAT3-COL4A2) expression profile informed by the networks was able to discriminate two hypertrophic cardiomyopathy patients with extreme fibrosis phenotypes. Patient-specific network features also associate with other important hypertrophic cardiomyopathy clinical phenotypes. These proof-of-concept findings introduce personalized protein-protein interaction networks (reticulotypes) for characterizing patient-specific pathobiology, thereby offering a direct strategy for advancing precision medicine.

## Abstract (web summary)

Understanding patient-specific pathobiological pathways is a critical step for advancing precision medicine. Here the authors show that individualized protein-protein interaction networks provide key insight on patient-level pathobiology and clinically relevant pathophenotypic characteristics in a complex disease.

## Introduction

Pursuing patient-specific diagnostic and therapeutic strategies defines contemporary medicine^1^. This goal has particular importance in cardiovascular diseases owing to variability in key pathogenetic mechanisms among patients with the same diagnosis, hampering therapeutic optimization clinically. Focusing on rare genetic variants alone or on simple fold-change in transcriptomic data between patient groups, whereas helpful, may overlook important and integrative determinants of complex cardiovascular pathophenotypes^2^. Alternative strategies that individualize knowledge based on functionally relevant biological pathways unique to individual patients may provide critical insight toward achieving the goals of precision medicine.

Hypertrophic cardiomyopathy (HCM) is defined principally by left ventricular (LV) hypertrophy without increased cardiac afterload or another underlying pathophysiological etiology, and causes heart failure at any age^3^. Familial inheritance and LV hypercontractility observed in some HCM patients have been ascribed classically to variants in genes encoding cardiomyocyte sarcomere proteins^4^; however, HCM is a heterogeneous disease that involves numerous sarcomere-independent morphological features, present to varying degrees in individual patients. A contemporary view of the HCM spectrum suggests that multiple endophenotypes converge in a unique pattern to generate patient-specific clinical phenotypes^5^. Thus, reductionist methods may be limited for personalizing the pathobiological basis of HCM, as well other complex diseases.

Network medicine integrates functionally important protein–protein interactions (PPIs), and has been used to identify critical pathways that regulate endophenotypes of relevance to HCM (e.g., fibrosis)^6,7^. However, prior PPI networks have not incorporated patient-level variability in pathobiology as reflected in individualized differences in gene expression, and, thus, may have limited applicability to patients with extreme phenotypes. Recently, we proposed the concept of individualized disease modules in which differential gene expression and genetic variants are incorporated (individualized reticulotypes) as a strategy for exploiting the power of network medicine for precision medicine^8^. In this work, we show that individualized PPI networks provide critical insight into determinants of patient-specific and clinically relevant HCM pathophenotypic characteristics.

## Results

### Study population

Samples were received and processed for 18 HCM patients and 5 healthy controls; the clinical characteristics of each group are summarized in Supplementary Table 1 and described individually in Supplementary Data File 1. There was no significant difference in age between cohorts, although a majority of the HCM patients and controls were male and female, respectively. Trauma was implicated as a cause of death in 60% of the controls. The profile of HCM patients was as expected, including New York Heart Association Functional Class II or III, and increased maximal LV thickness (20 [18–22] mm), LV end-diastolic volume (178 ± 30.6 mL), LV end-systolic volume (59 ± 16 mL), and LV mass (147 ± 44 g). A minority of HCM patients in this study had a family history of HCM, sudden cardiac death, syncope, or non-sustained ventricular tachycardia on Holter monitoring.

### Transcriptomic profile of the control and HCM cohorts

The myocardial RNA-Seq raw count matrix included 44,285 genomic features from 23 samples with high replicate quality within HCM (*N* = 18) and control (*N* = 5) samples, inclusive of protein-coding genes, pseudogenes, long non-coding RNAs, and others (referred to collectively as genes in this report) (Supplementary Tables 2 and 3). The transcript alignment by Tophat2 was 90.3 ± 1.0% for the total cohort, with no significant differences observed between HCM and controls (90.0% vs. 91.7%, *P* = 0.49). Differentially expressed genes were analyzed to preview global differences in the molecular profile between HCM and controls. We identified 2238 significantly differentially expressed genes between the control and HCM cohorts (1649 are protein-coding genes); the expression pattern for individual patients is presented in heat map form in Supplementary Fig. 1a. Partitioning variance in gene expression by the first and second principal component vectors (28% and 19% of the total variance, respectively) demonstrated separation between patient groups, but also substantial separation among patients within the HCM group (Supplementary Fig. 1b). The coefficient of variance (CV) range for the expression of genes among HCM patients was 16–424%, with 6,785 (41%) genes having a CV ≥ 40% (Supplementary Fig. 1c). These collective data suggest important pathobiological variability across patients with the same HCM diagnosis, and are consistent with the clinical heterogeneity observed in this (and many other) study populations^5^.

### Individual-patient networks (reticulotypes)

**Fig. 1:**
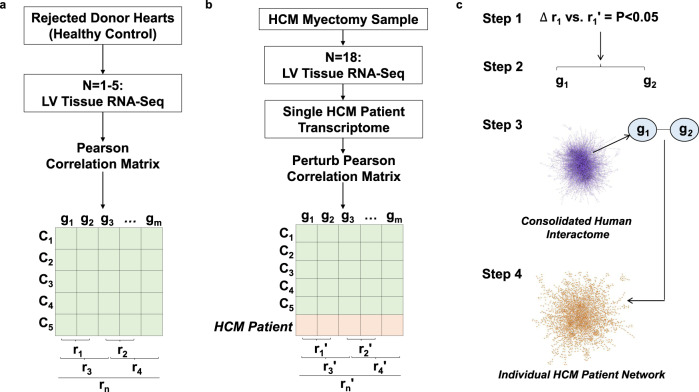
Strategy for developing patient-specific protein–protein interaction networks in HCM. **a** Left ventricular (LV) myocardial samples from rejected heart transplant donors serving as healthy controls (*N* = 5) (C_1_–C_5_) were analyzed using RNA-Seq, and the Pearson correlation coefficient (*r*) was calculated for all gene (*g*) pairs. n, total combination of pairwise correlations; m, total number of genes. **b** Anterior septal myectomy samples from hypertrophic cardiomyopathy (HCM) patients were analyzed using RNA-Seq. The transcriptomic profile of an individual HCM patient was added to the control gene expression matrix, and the new Pearson correlation coefficient (r′) was calculated for all gene pairs. The HCM patient transcriptomic profile was then removed from the matrix and the process was repeated sequentially for the HCM (*N* = 18). **c** (Step1) Statistically significant changes between the *r* and *r*′ coefficients were collected, and (Step 2) those gene pairs (g_1_–g_2_) were mapped to the consolidated human interactome (Step 3), which contains information on 15,489 proteins and 188,973 protein–protein interactions (PPIs). The statistical test used in this step was the two-tailed *Z*-test, and the *P* values were adjusted by the Bonferroni correction procedure for multiple comparison. In Step 4, gene pairs for which a PPI was identified in the consolidated human interactome were used to generate individual-patient PPI network reticulotypes.

There were 44,285 genomic features in the RNA-Seq data set. After removing those with zero counts in all of the five control samples, 26,946 genes remained. Next, we only kept genes with ≥10 counts in ≥1 healthy control sample(s), resulting in a set of 16,457 genes that was used for further analysis. The HCM patient networks were engineered by first establishing a Pearson’s correlation matrix inclusive of all transcriptomic data from the control patient cohort, which included 135,408,196 correlations among 16,457 genes (Fig. 1 and Supplementary Fig. 1d). The protein product of gene pairs whose correlations were significantly different following the addition of a single patient transcriptomic profile was mapped to the human interactome. Since the primary goal of this work was to study PPIs, only protein-coding genes were used to build the patient-specific networks.

**Fig. 2:**
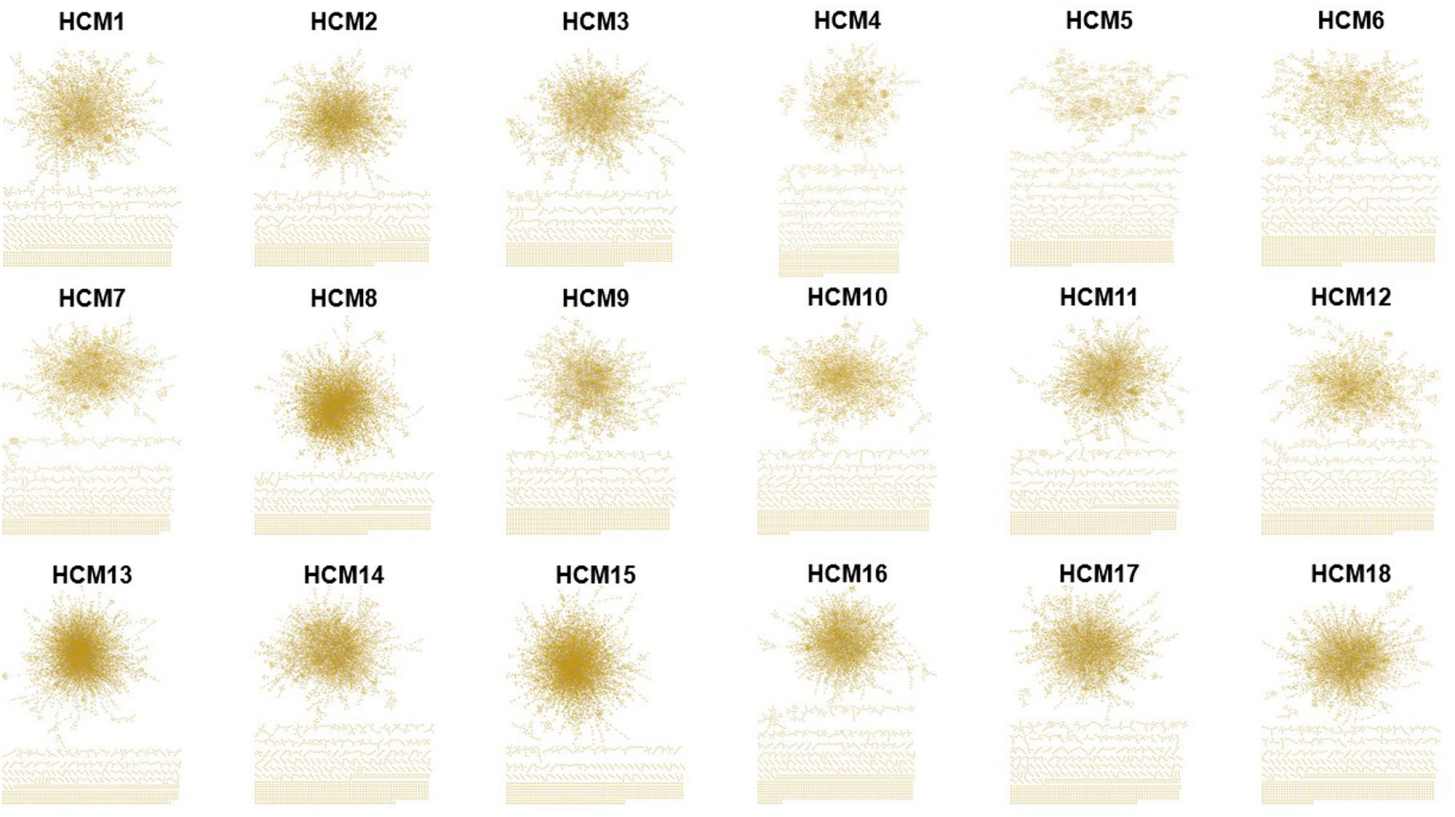
Individual protein–protein interaction networks (reticulotypes) for all HCM patients. The transcriptomic profile of anterior septal myectomy specimens from patients with hypertrophic cardiomyopathy (HCM) (*N* = 18) was analyzed using a two-step method that included a correlation matrix and protein–protein interaction (PPI) analysis from the consolidated human interactome, as outlined in Fig. 1. The derivative individualized patient networks varied in topology (median = 3629 [2474 min, 5031 max] number of nodes, representing proteins) and complexity (median = 3696 [2014 min, 6626 max] number of edges, representing links or physical protein–protein interactions between nodes), and were used in further analyses informing patient-specific HCM pathophenotypic and clinical parameters.

The resulting patient networks varied widely in topology (median = 3629 [2474 min, 5031 max] number of nodes) and complexity (median = 3696 [2014 min, 6626 max] number of edges) (Fig. 2). No significant differences in the number of significant gene pairs (*P* = 0.38), network nodes (*P* = 0.24), or network edges (*P* = 0.18) were observed by sex. A significantly greater overlap for nodes was observed compared with edges across the HCM networks (0.73 ± 0.04 vs. 0.48 ± 0.09, *P* = 3.2e-82) (Table 1). Density, diameter, heterogeneity, and other topological network features for each HCM patient are provided in Supplementary Table 4. The HCM5 network was smaller than the networks from other HCM patients. As such, the characteristic edge path length of 11.35 was an outlier (*P* = 0.02); however, no other parameter was significantly different compared to the remainder of the cohort. As an alternative filter (i.e., robustness test) for preparing the networks, we kept only those genes with ≥10 read counts in all samples from the healthy cohort, which led to 12,305 genes for further analyses. However, overlap between networks from the original vs. revised filter methodology was 97.3 ± 0.3% and 96.3 ± 0.28% for nodes and edges, respectively, suggesting nominal differences in network topology between these different filters (Supplementary Table 5).

**Table 1:** Node and edge overlap between individualized patient HCM networks.

Node overlap between individual-patient HCM networks																																				
	HCM 1		HCM 2		HCM 3		HCM 4		HCM 5		HCM 6		HCM 7		HCM 8		HCM 9		HCM 10		HCM 11		HCM 12		HCM 13		HCM 14		HCM 15		HCM 16		HCM 17		HCM 18	
HCM 1	1		0.75		0.60		0.72		0.73		0.73		0.78		0.76		0.72		0.70		0.64		0.72		0.79		0.70		0.80		0.74		0.77		0.72	
HCM 2			1		0.64		0.71		0.74		0.76		0.71		0.75		0.78		0.73		0.67		0.68		0.78		0.68		0.76		0.69		0.70		0.69	
HCM 3					1		0.59		0.63		0.63		0.64		0.75		0.61		0.61		0.64		0.59		0.76		0.67		0.71		0.64		0.64		0.67	
HCM 4							1		0.64		0.63		0.80		0.77		0.63		0.65		0.60		0.83		0.76		0.70		0.85		0.81		0.79		0.66	
HCM 5									1		0.66		0.76		0.78		0.69		0.73		0.68		0.72		0.83		0.77		0.79		0.77		0.78		0.75	
HCM 6											1		0.79		0.77		0.66		0.69		0.66		0.74		0.82		0.74		0.79		0.76		0.79		0.72	
HCM 7													1		0.74		0.75		0.72		0.64		0.79		0.80		0.71		0.81		0.78		0.79		0.70	
HCM 8															1		0.76		0.75		0.79		0.74		0.71		0.75		0.70		0.73		0.74		0.74	
HCM 9																	1		0.75		0.65		0.69		0.82		0.71		0.77		0.73		0.74		0.73	
HCM 10																			1		0.65		0.65		0.83		0.72		0.76		0.71		0.71		0.74	
HCM 11																					1		0.58		0.78		0.69		0.74		0.64		0.65		0.71	
HCM 12																							1		0.78		0.71		0.85		0.81		0.82		0.67	
HCM 13																									1		0.83		0.72		0.79		0.78		0.81	
HCM 14																											1		0.75		0.71		0.71		0.71	
HCM 15																													1		0.80		0.82		0.73	
HCM 16																															1		0.75		0.66	
HCM 17																																	1		0.67	
HCM 18																																			1	

Edge overlap between individual-patient HCM networks																																				
		HCM 1		HCM 2		HCM 3		HCM 4		HCM 5		HCM 6		HCM 7		HCM 8		HCM 9		HCM 10		HCM 11		HCM 12		HCM 13		HCM 14		HCM 15		HCM 16		HCM 17		HCM 18
HCM 1		1		0.56		0.32		0.54		0.56		0.57		0.59		0.44		0.53		0.50		0.37		0.51		0.50		0.42		0.51		0.52		0.56		0.48
HCM 2				1		0.32		0.47		0.53		0.59		0.49		0.40		0.60		0.56		0.39		0.46		0.49		0.39		0.46		0.41		0.45		0.42
HCM 3						1		0.30		0.36		0.36		0.32		0.40		0.33		0.32		0.35		0.26		0.39		0.31		0.32		0.29		0.30		0.36
HCM 4								1		0.45		0.44		0.65		0.46		0.42		0.39		0.30		0.73		0.45		0.41		0.66		0.66		0.61		0.38
HCM 5										1		0.47		0.57		0.48		0.51		0.52		0.43		0.52		0.58		0.54		0.52		0.56		0.59		0.52
HCM 6												1		0.62		0.47		0.48		0.49		0.42		0.56		0.56		0.51		0.54		0.56		0.61		0.50
HCM 7														1		0.42		0.55		0.51		0.33		0.67		0.55		0.46		0.57		0.61		0.65		0.46
HCM 8																1		0.43		0.43		0.51		0.44		0.37		0.42		0.33		0.41		0.41		0.43
HCM 9																		1		0.56		0.38		0.46		0.55		0.41		0.47		0.48		0.52		0.48
HCM 10																				1		0.40		0.41		0.58		0.44		0.45		0.45		0.47		0.51
HCM 11																						1		0.29		0.48		0.38		0.37		0.31		0.32		0.44
HCM 12																								1		0.53		0.47		0.68		0.67		0.69		0.42
HCM 13																										1		0.66		0.37		0.54		0.52		0.58
HCM 14																												1		0.45		0.48		0.47		0.48
HCM 15																														1		0.57		0.61		0.40
HCM 16																																1		0.57		0.40
HCM 17																																		1		0.40
HCM 18																																				1

The transcriptomic profile of anterior septal myectomy specimens from patients with hypertrophic cardiomyopathy (HCM) (*N* = 18) was analyzed using a two-step method that included a correlation matrix and protein–protein interaction analysis resulting in individualized patient networks. Nodes (representing proteins in the networks) common to individualized patient network pairs are presented in the top panel, and edges (representing interactions between proteins in the networks) common to individualized patient network pairs are presented in the bottom panel. 1, full overlap; 0, no overlap.

### Individual-patient networks delineate the molecular profile of cardiomyopathies

Genetic, transcriptomic, and endophenotype overlap is reported between different cardiomyopathies, despite divergent phenotypes^2,9^. Thus, we next aimed to determine whether our network analysis could discriminate the transcriptomic profile of patients with HCM from dilated cardiomyopathy (DCM). Gene expression profiles from patients with DCM compared with controls^10^ were used to generate individual DCM patient networks as was done for HCM. Compared with the HCM networks, the DCM networks were larger (median = 3718 ± 722 vs. median = 4676 ± 731 number of nodes, *P* = 0.01) and generally included more edges (median = 3696 [3164 min – 4342 max] vs. median = 4880 [3880 min – 6417 max] number of edges, *P* = 0.057) (Supplementary Table 6). Node overlap between HCM and DCM was 0.55 ± 0.03; however, overlap among network edges between these two diseases was 0.03 ± 0.0 (*P* < 2.2E-16 for node vs. edge overlap) (Table 2, Supplementary Tables 7 and 8). Additional network topology features and statistical comparisons between the HCM vs. DCM cohorts are reported in Supplementary Table 4. Findings using our methodology when comparing network node and edge overlap between the  HCM cohort vs. a separate DCM cohort are presented in Supplementary Data Files 2, 3; *P* < 2.2E-16 for node vs. edge overlap). When constructing individual-patient PPI networks from publicly available RNA-Seq database, data from similar analyses are also presented between other (i.e., validation) HCM vs. DCM cohorts in Supplementary Data Files 4, 5 (*P* < 2.2E-16 for node vs. edge overlap) and Supplementary Data Files 6, 7 (*P* < 2.2E-16 for node vs. edge overlap).

**Table 2:** Node and edge overlap between individual-patient networks for the HCM and DCM cohorts.

	Node overlap						Edge overlap					
	DCM1	DCM2	DCM3	DCM4	DCM5	DCM6	DCM1	DCM2	DCM3	DCM4	DCM5	DCM6
HCM1	0.48	0.47	0.51	0.65	0.56	0.62	0.03	0.02	0.03	0.04	0.03	0.04
HCM2	0.48	0.46	0.51	0.65	0.56	0.62	0.03	0.03	0.03	0.04	0.03	0.04
HCM3	0.48	0.47	0.53	0.66	0.57	0.62	0.02	0.02	0.02	0.04	0.03	0.04
HCM4	0.49	0.47	0.52	0.65	0.57	0.61	0.02	0.02	0.02	0.04	0.03	0.03
HCM5	0.49	0.48	0.54	0.65	0.57	0.63	0.03	0.03	0.03	0.04	0.03	0.04
HCM6	0.48	0.48	0.52	0.65	0.57	0.61	0.03	0.03	0.02	0.05	0.04	0.04
HCM7	0.47	0.46	0.51	0.64	0.55	0.60	0.02	0.02	0.03	0.04	0.03	0.03
HCM8	0.57	0.57	0.56	0.61	0.55	0.59	0.04	0.04	0.04	0.04	0.04	0.04
HCM9	0.48	0.48	0.53	0.65	0.57	0.62	0.03	0.03	0.03	0.04	0.04	0.04
HCM10	0.48	0.47	0.52	0.65	0.57	0.63	0.03	0.02	0.03	0.04	0.04	0.04
HCM11	0.48	0.47	0.52	0.65	0.55	0.61	0.02	0.02	0.02	0.04	0.03	0.03
HCM12	0.48	0.46	0.50	0.64	0.55	0.60	0.02	0.02	0.03	0.04	0.03	0.03
HCM13	0.57	0.57	0.54	0.61	0.55	0.58	0.04	0.04	0.04	0.04	0.04	0.04
HCM14	0.47	0.48	0.50	0.62	0.53	0.59	0.03	0.02	0.03	0.04	0.03	0.03
HCM15	0.56	0.55	0.53	0.61	0.54	0.58	0.04	0.04	0.04	0.04	0.04	0.04
HCM16	0.46	0.46	0.49	0.63	0.54	0.59	0.02	0.02	0.03	0.05	0.03	0.04
HCM17	0.47	0.46	0.50	0.63	0.54	0.60	0.02	0.02	0.03	0.04	0.04	0.04
HCM18	0.47	0.48	0.51	0.63	0.54	0.60	0.03	0.03	0.03	0.04	0.03	0.03

The transcriptomic profile of anterior septal myectomy specimens from patients with hypertrophic cardiomyopathy (HCM) (*N* = 18) was analyzed using a two-step method that included a correlation matrix and protein–protein interaction analysis, resulting in individualized patient networks. The same analytical method was used to develop individualized networks for patients with dilated cardiomyopathy (DCM) (*N* = 6) using a transcriptomic dataset published previously^10^. Nodes (representing proteins in the networks) and edges (representing links between proteins in the networks) common to individualized patient HCM vs. DCM network pairs are presented. 1, full overlap; 0, no overlap.

We next tested the hypothesis that in the human interactome, HCM and DCM are likely to be close to each other owing to (some) common pathogenetic genes (represented by nodes), but distinct by virtue of few overlapping PPIs (represented by edges). Among HCM (*N* = 112) and DCM (*N* = 155) disease genes collected from Phenopedia, we identified *N* = 50 that were common to both diseases (*P* < 2.1e-72). This dataset was mapped to the human interactome, resulting in a network that included discrete HCM and DCM disease modules, as well as nodes that overlap between HCM and DCM (Supplementary Fig. 2a). We then analyzed, within the interactome, the topological relationship of all individual-patient networks in this study. This step provided an opportunity to contextualize, topologically, the HCM and DCM patient-specific disease networks relative to their respective disease modules (from Supplementary Fig. 2a). The results of this analysis are provided in Supplementary Fig. 2b and depict the divergence between HCM patient-specific networks and DCM patient-specific networks with respect to network edges (PPIs).

We next tested the postulate that sarcomere protein-coding genes harboring pathogenic variants would be central to PPIs in individual HCM patient networks. Among the 17 reported putative HCM-causing genes^11,12^ included among 774 pathogenic and 475 likely pathogenic variants, there were four variants in two HCM genes affecting a total of four patients (*N* = 25%): three patients had a pathogenic mutation (MYBPC3: c.1928-2 A > G; MYBPC3: c.G2497A:p.A833T; MYL2: c.C141A:p.N47K) and one patient had a likely pathogenic mutation (MYL2: c.G2429A:p.R810H) (Supplementary Table 9). However, nodes corresponding to these genes were not present in any of the individualized PPI networks from these four patients. The expression pattern for the 17 HCM-causing genes is presented in Supplementary Fig. 3.

### Using individualized PPI networks to understand endophenotype enrichment in HCM

As the principal clinical feature of HCM is LV hypertrophy, we next performed an endophenotype enrichment analysis to identify hypertrophy signaling pathway enrichment in the individual networks. Unexpectedly, hypertrophy signaling was not included among the 30 endophenotypes that were significantly enriched across the patient networks for the entire HCM (and DCM) cohort according to analyses using the hypergeometric test (Supplementary Fig. 4a and Supplementary Data Files 8–10). Increased fibrotic matrix remodeling is reported in early-era HCM autopsy series^13^, implicated indirectly by modern-day imaging as a determinant of cardiac structural remodeling^14^, and emerged as important in our endophenotype analysis of individual HCM networks. Indeed, a significantly greater proportion of HCM patient networks was enriched for fibrosis compared with hypertrophy pathways (94% vs. 11%, 1.3e-05, *N* = 18) (Supplementary Fig. 4b, Supplementary Table 10). Compared with controls, increased interstitial collagen by trichrome staining was evident in the HCM myectomy samples (4.7 ± 0.42 vs. 14.3 ± 0.02% collagen/high powered field, *P* = 0.04, *N* = 5 controls, and *N* = 18 HCM patients), but no significant difference in cardiomyocyte cross-sectional diameter was observed (Supplementary Fig. 4c, d). Importantly, no patient in the HCM cohort had any of the three commonly cited putative HCM fibrosis gene variants: αMHC Arg403Gln, αMHC Arg719Trp, or cTnt^Q92^^15^.

### JAK2-STAT3-COL4A2 expression differentiates extreme fibrotic HCM phenotypes

There were 41 fibrosis nodes common to all HCM patient networks, and we identified 4 (9.7%) of them whose genes were differentially expressed significantly compared to controls: hypoxia-inducible factor (HIF)-1α, insulin growth factor receptor-1 (IGFR-1), Janus kinase (JAK)1, and JAK2 (Supplementary Table 11 and Supplementary Fig. 5). Of these, HIF-1α and IGFR-1 have already been reported in HCM^16–18^. Compared with JAK1, upregulation of IL-6 is associated more strongly with JAK2, and IL-6 is increased in HCM^19^. Furthermore, prior reports implicate increased JAK2 bioactivity in hypertrophic heart disease diagnostic of HCM^20,21^, but data on JAK2 in confirmed HCM cases are lacking. Therefore, we focused next on JAK2 signaling in our study.

**Fig. 3:**
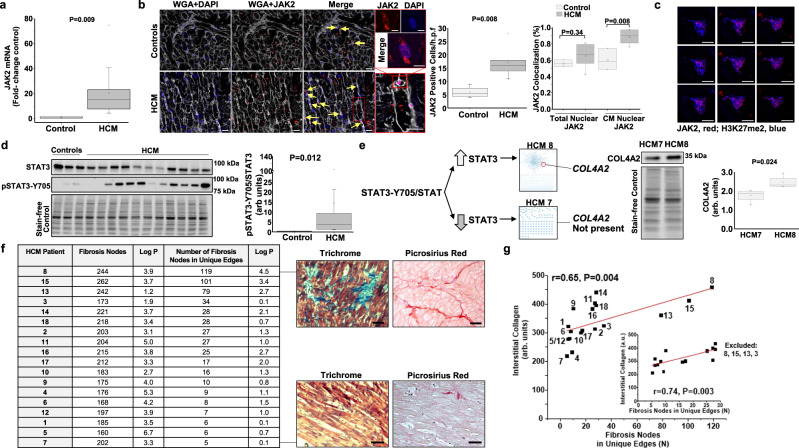
Individual-patient network features inform the HCM fibrosis pathophenotype. Janus kinase-2 (JAK2) was one of four profibrotic nodes differentially expressed between HCM and control patients that was also present in all HCM patient networks. **a** In left ventricular (LV) homogenates, JAK2 mRNA quantity was increased significantly in HCM (*N* = 16) compared with control (*N* = 3), **b** which was consistent with anti-JAK2 immunofluorescence demonstrating increased JAK2-positivity of cells (yellow arrows), **c** as well as increased nuclear JAK2 expression in cardiomyocytes (supported by Z-stack imaging) (*N* = 3 for controls; *N* = 7 for HCM). WGA, wheat germ agglutinin; H3K27me2, methylated lysine 27 of histone H3, and DAPI show nuclear signal. Scale bar = 20 µm, and 6 µm for inset/Z-stack images. **d** Increased LV homogenate p-STAT3-Y705/total STAT3 was observed in HCM (*N* = 11) compared with control (*N* = 3). HCM patients 8 and 7 had the greatest and least p-STAT3-Y705/total STAT3 ratio, respectively, and the STAT3 collagen target COL4A2 was present in the HCM8 network, but absent from the HCM7 network. **e** Analysis of collagen gene variants suggested a particular difference between HCM8 and HCM7 for COL4A-218, which was confirmed by immunoblot. Immunoblot images are within the same blot, but cropped and merged (*N* = 4 samples/condition). **f** The total number of fibrosis nodes and the number (*N*) of fibrosis nodes in edges unique to a single HCM patient network were calculated. Histopathology confirmed that HCM8 and HCM7 represent extreme fibrosis pathophenotypes. Scale bar = 50 µm. **g** The relationship between the number of unique fibrosis edges for each patient network and interstitial collagen quantity by trichrome stain, which was maintained after excluding the most densely connected patient networks (*N* = 4 removed from analysis, shown in inset). The HCM patient number is provided with each datapoint. Representative micrographs and blots are shown. **a**–**e**, **g** Student’s unpaired two-tailed *t* test or Mann–Whitney tests were used as appropriate. **a**, **b**, **d**, **e** box plots: mean (square), median (horizontal line), interquartile range (box distribution), and maximum and minimum (stars). **f** The one-tail hypergeometric test was used to generate the log *P* values. **g** For linear regression, Pearson’s correlation coefficient is presented. Source data are available.

Compared with control LV homogenates, JAK2 mRNA quantity was increased in HCM by 11.5-fold (*P* = 0.012, *N* = 3 controls, and *N* = 16 HCM patients) (Fig. 3a), which was directionally similar to findings for JAK2 expression in paraffin-embedded heart sections from the same patients (6.0 ± 1.1 vs. 17 ± 2.4 JAK2-positive cells, *P* = 0.008, *N* = 4 controls, and *N* = 6 HCM patients) (Fig. 3b). Increased localization of JAK2 to the cardiomyocyte nucleus was suggested by findings from samples co-stained for JAK2 and DAPI or H3K27me2, and analyzed by confocal microscopy and Z-stack imaging (Fig. 3c). Nuclear expression of JAK2 is associated with upregulated JAK2-STAT3 signaling^22^, and increased STAT3 has been shown in HCM in vivo^23^. Thus, we next measured total STAT3 and its activated form, p-STAT3-Y705, by immunoblot. Compared with controls, p-STAT3-Y705/STAT3 was increased significantly in HCM (0.3 [0.2–0.3] vs. 3.7 [1.2–9.3] a.u., *P* = 0.013, *N* = 3 controls, and *N* = 11 HCM patients) (Fig. 3d), with HCM patient eight (HCM8) and HCM7 expressing the greatest and least difference in P-STAT-Y705/STAT3, respectively, compared with control (21.2-fold vs. 1.01-fold increase over control).

A central goal of these experiments was to determine whether features from patient-specific networks could inform the fibrotic pathophenotype in individual patients. Therefore, we hypothesized that the HCM8 and HCM7 patient networks were distinguishable by collagen IV (COL4), which is a basal lamina protein implicated in interstitial fibrosis and regulated by STAT3 in profibrotic cell types^24^. Consistent with this hypothesis, the COL4 isoform COL4A2 was present in the HCM8 but not HCM7 networks. From this analysis, we identified a 2.03-fold increase in the myectomy gene expression of the splice variant COL4A-218 in HCM8 vs. HCM7 (4227 vs. 2081 read counts), which was directionally consistent with a significant 32% (*P* < 0.03, *N* = 3) increase in COL4A2-218 expression by immunoblot (Fig. 3e). Furthermore, HCM8 and HCM7 had the greatest and least quantity of interstitial fibrosis, respectively, across the entire HCM cohort (458 ± 3.8 vs. 143 ± 5.3 arb. units, *P* < 0.0001, *N* = 3). COL4A2 was also identified in the HCM18, but not HCM5, patient networks, which were patients with the second most and second least extent of cardiac fibrosis, respectively. In Fig. 3f, fibrosis network features for each HCM patient are provided, of which HCM8 and HCM7 were ranked highest and lowest, respectively. The correlation between fibrosis network features and collagen quantity across all HCM samples is shown in Fig. 3g.

### Network features unique to each patient inform the clinical phenotype features of HCM heart failure

**Fig. 4:**
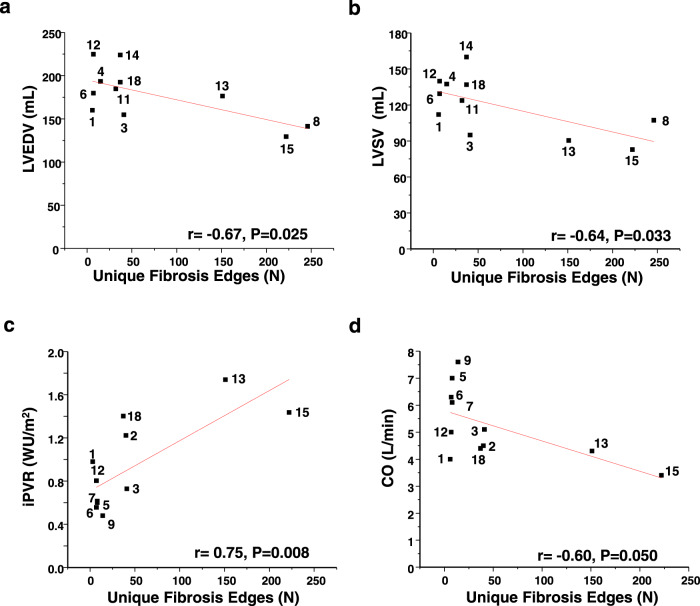
Number of unique fibrosis edges informs predictive physiological parameters in HCM heart failure clinically. The number of unique fibrosis edges, defined as the number (N) of edges unique to a single HCM patient network that also involve at least one fibrosis gene, was calculated. **a**–**c** The number of unique fibrosis edges correlated inversely with decreasing left ventricular end-diastolic volume (LVEDD) (*N* = 11) and left ventricular stoke volume (LVSV) (*N* = 11), and positively with indexed pulmonary vascular resistance (iPVR) (*N* = 11), which are pathological, morphological, functional, and hemodynamic changes, respectively, that promote HCM heart failure. **d** Furthermore, the number of unique fibrosis edges correlated inversely with cardiac output (*N* = 11), suggesting, overall, that heart failure severity in this HCM study cohort was associated with the number of unique fibrosis edges on an individual-patient level. CO, cardiac output; WU, Wood unit. **a**–**d** Pearson’s and correlation analysis was performed. For **a**–**d**, the HCM patient number is provided with each datapoint. Source data are available.

The individual-patient HCM networks inclusive of only edges unique to each patient varied in topology (505 [83 min, 1880 max] number of nodes) and complexity (385 [42 min, 1722 max] number of edges) (Supplementary Fig. 6, Supplementary Table 12). The average number of fibrosis nodes, and the number of fibrosis nodes in edges unique to a single HCM patient network were 202 ± 6.68 (log *P* = 3.7 ± 0.3) and 31 ± 7.9 (log *P* = 1.5 ± 0.3), respectively, suggesting variability in fibrosis burden across HCM patients in silico. A finding in earlier analyses was that the PPI network connectivity pattern distinguished patients effectively; therefore, we focused next on the relevance of the number of unique fibrosis edges to interstitial collagen and measures of heart failure progression in HCM. Unique fibrosis edges are the edges specific to a single HCM patient network that also involved at least one fibrosis gene. Indeed, the number of unique fibrosis edges correlated inversely with LV end-diastolic volume (*r* = −0.67, *P* = 0.025) and LV stroke volume (*r* = −0.64, *P* = 0.033), and positively with indexed pulmonary vascular resistance (*r* = 0.76, *P* = 0.010) (Fig. 4a–c). These data imply abnormalities in LV structure, LV function, and cardiopulmonary hemodynamic variables related to HCM heart failure as a function of the number of unique fibrosis edges (a proxy for the molecular drivers of the extent of fibrosis), providing the basis for next analyzing cardiac output (CO). An inverse association between the network-based determinants of the extent of fibrosis and CO (*r* = −0.60, *P* = 0.050) was observed (Fig. 4d), suggesting collectively that fibrosis pathway topology is associated with key HCM heart failure clinical parameters.

## Discussion

In this study, transcriptomic data from rare myectomy samples were used to develop HCM patient-specific PPI networks. Partial overlap was observed for the content of each HCM network; however, major differences in network connectivity reflecting differences in gene expression emerged unexpectedly. Individual-patient PPI network topology distinguished HCM from DCM in silico, a finding validated in separate cohorts, suggesting that unique PPIs may be important for understanding biological and phenotypic variability across different cardiomyopathies. As a mechanism-based proof-of-principle, we show that STAT3-COL4A2 expression, informed by the patient-specific networks, corresponded to variability in HCM fibrosis phenotypes among patients within the study cohort. Fibrosis features unique to each patient network were also associated with cardiac morphological and hemodynamic parameters that are important clinically in HCM. Overall, our findings suggest that a pathway-centered analysis of individual differences in patient-specific PPI networks helps decipher the reticulotypes in HCM and their pathobiological consequences.

Widespread availability of high-throughput multiplex array platforms has expanded access to vast amounts of data in disease populations. Importantly, however, extrapolating the biological importance of a particular gene-based solely on its expression, or assuming disease causality from a single hereditary event, may be misleading or overlook integrated pathways that determine complex phenotypes. Furthermore, classical summary statistics focusing on averages to compare the profile of patient groups (e.g., HCM vs. control) may mask important biological diversity: in this study, wide heterogeneity in the transcriptomic profile was observed across an 18-patient HCM cohort, akin to the findings reported for other diseases predisposing to heart failure, including myocardial infarction and pulmonary hypertension as two examples^25,26^. Nonetheless, strategies that offset these limitations and, instead, focus on functionally important features on an individual patient-level have been lacking^9^.

We used a two-step strategy in which the functional significance of information gleaned from a sample-specific correlation matrix^27^ was established using the consolidated human interactome. The importance of our network approach was strengthened by sharp distinctions in the PPI connectivity pattern of HCM relative to DCM, despite modest overlap in the content (e.g., proteins) of these networks. This observation, affirmed by analyses localizing individual-patient networks from this study to the human interactome, provides context to prior reports implicating pathogenicity of the same genetic variants in both HCM and DCM, such as *MYH6* and *ACTN2*, among others^28^, despite the fact these two phenotypes are grossly dissimilar. Findings in this study are directly in line with that postulate, as similarities were observed in the transcriptomic profiles of HCM and DCM viewed on average across the study cohorts. In contrast to this conventional approach, we rigorously explored the variability (genomic network diversity or biological noise) in the study cohorts in order to identify molecular determinants of individual pathobiologies (pathophenotype diversity). Thus, our data suggest that PPIs and their (unique) network context themselves provide crucial information ultimately needed to reconcile the divergent biofunctionality of a single biological component (e.g., protein) in two different pathophenotypes or between patients with the same pathophenotype.

Classically, HCM has been described as a monogenic disorder owing to one of >1400 variants in 11 genes encoding cardiomyocyte sarcomere proteins^5^. In this study, 25% of the HCM cohort had a putative pathogenic HCM variant, akin to findings from other cross-sectional HCM reports^29^. Importantly, contemporary-era population genetics data have revealed discordance between HCM prevalence and pathogenic genotype frequency^12,30^. In this study, the gene expression pattern for putative HCM-causing variants did not differ substantially for patients harboring those variants compared to other HCM patients. Furthermore, the PPI networks for patients with a putative HCM-causing variant were dense, but did not, in fact, include these respective proteins. These points taken together reinforce the importance of widening the spectrum of HCM determinants to factors beyond solely putative causative gene variants; genomic and network context are key determinants of clinical phenotype and its individualized features.

The endophenotype enrichment findings from patient networks in this study are consistent with reports implicating hypoxia- and redox-dependent post-transcriptional pathobiological mechanisms in HCM^31,32^. However, our data expand the number of endophenotypes previously unrecognized in HCM, particularly neoplastic- and DNA repair signaling, whereas refocusing importance on the role of classical HCM features that contribute to phenotypic heterogeneity. In particular, it has long been recognized that increased matrix expansion occurs due to interstitial collagen deposition^13^, and here we show that variable fibrosis gene expression patterns in HCM in silico were matched to specimen histopathology. In turn, neither enrichment in hypertrophic signaling determinants nor substantial cardiomyocyte enlargement were observed in this cohort. This finding illustrates a potentially underrecognized distinction between HCM-specific LV remodeling that is principally fibrosis-dominant in many patients and LV hypertrophy that occurs in other diseases due to increased afterload.

To validate our in silico findings, we first explored profibrotic network targets that were common to all HCM patients, including the protein tyrosine kinase JAK2, as well as IgF1R and HIF-1α. It was notable to us that the JAK2-V617F somatic mutation promotes overactivation of JAK2 signal transduction in myeloproliferative diseases^33^ and has been reported in a suspected case of HCM^20^, leading us to focus further on JAK2. We observed that transcriptional regulation of *JAK2* in proven HCM (in the absence of JAK2-V617F) corresponded to increased global LV and cardiomyocyte nuclear JAK2 expression, as well as activation of its downstream canonical target, STAT3. Increased IL-6 and oxidant stress, both reported in HCM^19,32^, regulate JAK2 bioactivity providing a plausible explanation for the acquired JAK2 upregulation in this disease. Non-fibrillar collagen isoforms including collagen type IV are implicated increasingly in extracellular matrix remodeling in cardiomyopathies^34^. Expression of COL4A2 is increased in JAK2-V617F polycythemia vera patients^20^, and *COL4* is regulated by STAT3 in profibrotic cell types^24^. This pathway, which has been evaluated indirectly in HCM previously^23^, was exploited here to discern heterogeneity in the pathophenotype between two patients with the greatest (HCM8) and least (HCM7) cardiac fibrosis endophenotypes, which was based on consistency across network, ex vivo, and histopathological analyses.

A minority of fibrosis nodes were common to all patient networks; thus, the relevance of this observation was explored using physiological parameters that risk-stratify HCM heart failure patients clinically^35,36^. The number of unique fibrosis edges (or specific PPIs) as a surrogate for the extent of network-driven fibrosis, which was variable across the HCM networks, was associated with LV end-diastolic volume, LV stroke volume, indexed pulmonary vascular resistance, and CO. Therefore, an additional major finding from this study was that linking PPI networks to clinically important data formed a novel, albeit preliminary, framework by which to bridge biological data with measurements used in patient care through instructive, targeted, and unbiased principles. Taken together with data suggesting that a personalized network may inform pathobiological differences (i.e., STAT3 and cardiac fibrosis), this work provides an incremental, but critical, step toward a PPI-based approach for individualized phenotyping.

Although sound gene ontology classification methods were used, it is possible that recently reported disease genes were not included in this study. Specifically, we used Phenopedia to collect endophenotype genes, recognizing that the true completeness of this database is not known. Using alternative gene sets^37^ should also be considered in future efforts to maximize generalizability of results. Furthermore, diverse biofunctionality is reported for many genes that emerged from our analyses, including JAK2, which was identified in 72% of all HCM endophenotypes. This finding limits the specificity of JAK2 to the pathogenesis of HCM fibrosis, but also supports the fundamental finding that PPI network edges more so than specific nodes are important for defining pathobiological differences between a cohort of patients with the same disease (i.e., HCM). Nonetheless, we recognize that alternative mechanisms to JAK2-STAT3 are anticipated to regulate HCM fibrosis, which may include established pathways, such as endothelin-1 and transforming growth factor (TGF)-β signaling, in particular^38^, as 17 of 18 (94%) of the HCM PPIs networks included one of these intermediaries. Similarly, targets beyond COL4A2, such as WWP2, shown previously to regulate cardiac fibrosis^39^ and identified in all 18 HCM patient networks, are anticipated to regulate HCM fibrosis. Therefore, detailed experiments are needed to establish the molecular mechanism(s) by which JAK2-STAT3 and other candidate pathways from the networks control COL4A2 or other collagens in individual patients.

The control  networks used here were derived from LV-free wall transcriptomic data from a largely female control population with prior drug/toxin exposure, as well as from the human interactome, which is vast but incomplete^40^. These confounders as well as potential batching effects may have affected the topology of individual HCM networks, and should be considered in further confirmatory studies. The timing of surgical myectomy is driven largely by symptoms, which do not correlate well with central hemodynamics in HCM despite their prognostic importance. Therefore, selection bias in the acquisition of samples may have affected our results from linear regression analyses comparing network fibrosis features with clinical parameters. In addition, expanding the study population is likely to diversify the results, particularly given the clinical heterogeneity in HCM. Mapping using T1-weighted cardiac magnetic resonance imaging might have provided data on interstitial fibrosis in clinically relevant terms^14^, but was not available in this study.

In conclusion, functional PPIs emerged as one strategy by which to delineate the molecular basis of HCM from DCM, which are two complex cardiovascular diseases with overlapping transcriptomic and endophenotypic profiles. Data from the personalized networks expand the range of molecular endophenotypes associated with HCM, but also may be used to informed patient-level differences in phenotype. As a proof-of-concept in support of this point, variability in JAK2-STAT3-COL4A2 biology informed by the PPI networks was used to predict the cardiac fibrosis phenotype of particular patients. Network features unique to each individual patient, individual reticulotypes, were also associated with parameters particularly relevant to heart failure and outcome in HCM, providing a potential link between individualized PPI networks and clinical phenotypes. Future work building on observations from this study are likely to advance precision medicine to a clinically relevant and readily applicable approach for HCM and many other complex phenotypes.

## Methods

### Study design

We tested the postulate that network medicine could be used to develop patient-specific PPI networks as a novel strategy by which to elucidate the pathobiology–pathophenotype relationship in complex diseases, focusing on obstructive HCM. All computational data included in the study were challenged with various sensitivity analyses to ensure internal consistency across iterations and dataset versions. Experimental data were reproduced across multiple iterations of the same experiment performed on different days (S.S., B.A.M.), and, whenever possible, replicated by different project investigators (E.A.). The number of patient samples included in this study was chosen based on availability and budgetary constraints. Nonetheless, in view of a priori knowledge that HCM is a heterogeneous disease clinically, and prior findings from multiplex data sets in HCM murine models^41^, we anticipated that a minimum of 10 patients was required to permit the emergence of informative differences in patient-specific PPI networks. Clinical data for patients was reviewed for accuracy independently by at least two board-certified cardiologists and HCM experts (M.S.M, E.J.R.). Upon optimizing the experimental methods, all data were included in the analyses unless a specific technical reason was present that confounded the interpretation of a finding. Whenever possible, interpretation of data by senior authors (B.A.M., B.J.M., J.L.) was blinded to the patient condition, and all authors had access to all primary data throughout the project.

### Study population

All patients with obstructive HCM referred for surgical septal myectomy at Tufts Medical Center (2009–2015) to treat symptomatic heart failure were available for analysis. The diagnosis of HCM was based on the standard definition, as reported previously^42^, requiring echocardiographic and/or cardiovascular magnetic resonance imaging evidence of hypertrophy (≥15 mm) in ≥1 LV segment occurring in the absence of a cardiac or systemic disease capable of causing the observed magnitude of LV hypertrophy (M.S.M, E.J.R, B.J.M.). Patient samples were selected at random. This study has been reviewed and approved by the Institutional Review Board (IRB) of Tufts Medical Center (#12019), Brigham and Women’s Hospital (#2015P001850), and the University of Utah (#30622). Informed consent was obtained from each patient at the time of surgery for the myectomy sample to be included in tissue and DNA banking for the purpose of future academic research studies.

Control samples were obtained from donors with non-failing hearts based on functional and structural evaluations (usually echocardiography and invasive hemodynamics), and no known cardiac disease in the past medical history. These control hearts had not been allocated for transplantation owing to non-cardiac reasons: donor size, donor malignancy, infectious disease risk, and other (S.G.D)^43^. Informed consent was obtained from the organ donor organization, Donor Connect.

### Building the individual-patient networks

We used network medicine to characterize individual HCM patients by virtue of functionally important PPIs. To accomplish this end, a gene correlation matrix was assembled from controls samples. This step allowed us to monitor more global changes in the gene profile caused by the addition of a single HCM patient dataset rather than overemphasizing changes in a select few mRNAs^9^.

We initiated the computational analysis by calculating the Pearson correlation coefficient (PCC) for each gene pair across all control samples (*N* = 5) (Fig. 1a). We then added an HCM sample, and recalculated the PCCs (Fig. 1b). If the change or perturbation of the PCC (i.e., ΔPCC) was significant according to pre-defined statistical thresholds^27^, we retained the gene pair. We repeated this calculation for each gene pair ad seriatim. After Bonferroni correction for multiple comparisons, we mapped significant, retained gene pairs to the human interactome (Fig. 1c). The interactome, which includes gene products (i.e., proteins), was consolidated from major publications on high-throughput, experimental protein–protein physical interactions^40,44–50^. The full details of resources can be found in the Supplementary Material. The human interactome we compiled has 15,489 proteins and 188,973 interactions.

Following this analysis, the PPIs in the human interactome that are retained correspond to significant gene pairs derived from the addition of each disease sample to the control gene expression matrix that perturb the control PCC (Fig. 1c)^27^. The resulting PPI network represents the dysfunctional or perturbed system in the corresponding patient, and, thus, can capture the underlying pathobiology accounting for phenotype heterogeneity and personalized information of individual patients across a cohort. We performed this analysis for each patient one-by-one to obtain individual networks for all patients. The same approach was utilized for a DCM cohort (*N* = 6) using RNA-Seq data published previously^10^. The network definition for unique edges is illustrated in Supplementary Fig. 1a. The overlap coefficient between networks for edges or nodes was calculated using the formula, $O\left( {A,B} \right) = \frac{{|A \cap B|}}{{\min (|A|,|B|)}}$, derived from the Jaccard index, defined as the size of the intersection between network A and network B divided by the size of the smaller network^51^. Networks were analysed and visualized using Cytoscape 3.5.1.

### Statistical methods

All statistical analyses were performed using Origin 9.01 or OriginPro, and GraphPad Prism v7.03. For analyses involving clinical variables, data are presented as mean ± SD or median [IQR] for normally and non-normally distributed data, respectively. Other data are presented as mean ± SEM unless otherwise indicated. Comparison between two groups was performed using the Student’s unpaired two-tailed *t* test. However, for analyses comparing the difference in overlap between network nodes vs. edges between cohorts, the Student’s paired *t* test was used. The Mann–Whitney and Kruskal–Wallis nonparametric tests were used to compare two or more non-normally distributed groups. The Pearson *r* and Spearman *ρ* coefficients are reported for linear regression analyses performed using data that were distributed normally and non-normally, respectively. The CV for the expression of genes was determined by calculating the ratio of standard deviation to mean-read count multiplied by 100. The hypergeometric test was used to determine if overlap among HCM and DCM genes in the interactome was significant. For analyses focusing specifically on differences in endophenotype enrichment between HCM and DCM cohorts, a hypergeometric test was used and data were corrected for multiple comparisons using the Benjamini–Hochberg procedure. The Grubb’s test was used to determine whether network topology parameters were outliers relative to the remaining HCM patient networks. Graphical representation of comparisons including *N* ≥ 3 uses the box plot format inclusive of mean (square), median (horizontal line), interquartile range (box distribution), and maximum and minimum (stars). Individual data points for analyses are provided in the Data Source File. A *P* value < 0.05 and false discovery rate < 0.05 were considered statistically significant, unless otherwise indicated.

### Reporting summary

Further information on research design is available in the Nature Research Reporting Summary linked to this article.

### Supplementary information

Supplementary Information

Description of Additional Supplementary Files

Supplementary Data 1

Supplementary Data 2

Supplementary Data 3

Supplementary Data 4

Supplementary Data 5

Supplementary Data 6

Supplementary Data 7

Supplementary Data 8

Supplementary Data 9

Supplementary Data 10

Reporting Summary

### Source data

Source Data

## Data Availability

An expanded methods section is available in the online Supplementary Information. The HCM RNA-seq data and HCM exome VCF files have been uploaded to the GEO database (accession ID: GSE160997). Other data that support the findings of this study are available from the corresponding author upon reasonable request. Source data are provided with this paper.

## Code availability

All of the source programming code used in this study has been uploaded to Gihub (https://github.com/bwh784/HCM [10.5281/zenodo.4429826])^52^.
