# Author Correction: Individualized interactomes for network-based precision medicine in hypertrophic cardiomyopathy with implications for other clinical pathophenotypes

**DOI:** 10.1038/s41467-022-34834-0

**Published:** 2022-11-17

**Authors:** Bradley A. Maron, Rui-Sheng Wang, Sergei Shevtsov, Stavros G. Drakos, Elena Arons, Omar Wever-Pinzon, Gordon S. Huggins, Andriy O. Samokhin, William M. Oldham, Yasmine Aguib, Magdi H. Yacoub, Ethan J. Rowin, Barry J. Maron, Martin S. Maron, Joseph Loscalzo

**Affiliations:** 1 Division of Cardiovascular Medicine, Department of Medicine, Brigham and Women’s Hospital and Harvard Medical School, Boston, MA USA; 2 Channing Division of Network Medicine, Department of Medicine, Brigham and Women’s Hospital and Harvard Medical School, Boston, MA USA; 3 Division of Cardiovascular Medicine, University of Utah School of Medicine, Salt Lake City, UT USA; 4 Nora Eccles Harrison Cardiovascular Research and Training Institute (CVRTI), University of Utah School of Medicine, Salt Lake City, UT USA; 5 Hypertrophic Cardiomyopathy Center, Cardiology Division, Tufts Medical Center, Boston, MA USA; 6 Division of Pulmonary and Critical Care Medicine, Department of Medicine, Brigham and Women’s Hospital and Harvard Medical School, Boston, MA USA; 7 Department of Cardiac Surgery, Imperial College of London, London, UK; 8 The Magdi Yacoub Heart Center, Aswan, Egypt

**Keywords:** Network topology, Cardiac hypertrophy, Molecular medicine

Correction to: *Nature Communications* 10.1038/s41467-021-21146-y, published online 08 February 2021

The original version of this Article contained an error in Fig. 4b, in which patient 4 was incorrectly labelled as Patient 3. The correct version of Fig. 4b is:
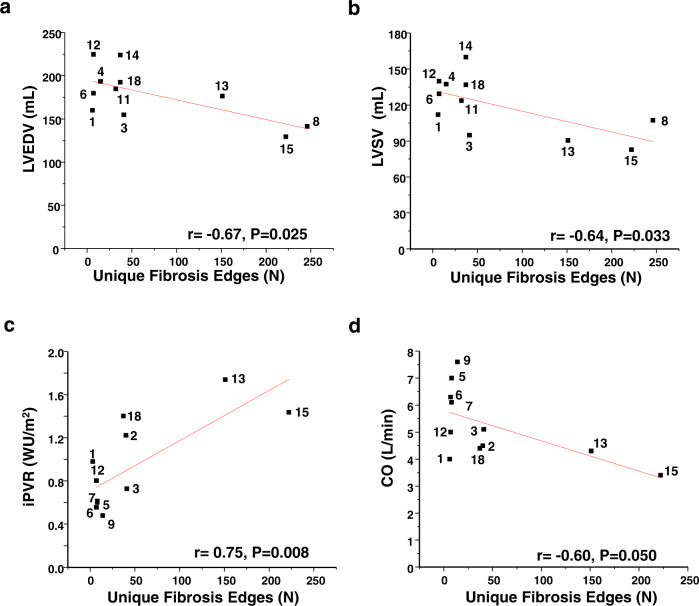


which replaces the previous incorrect version:
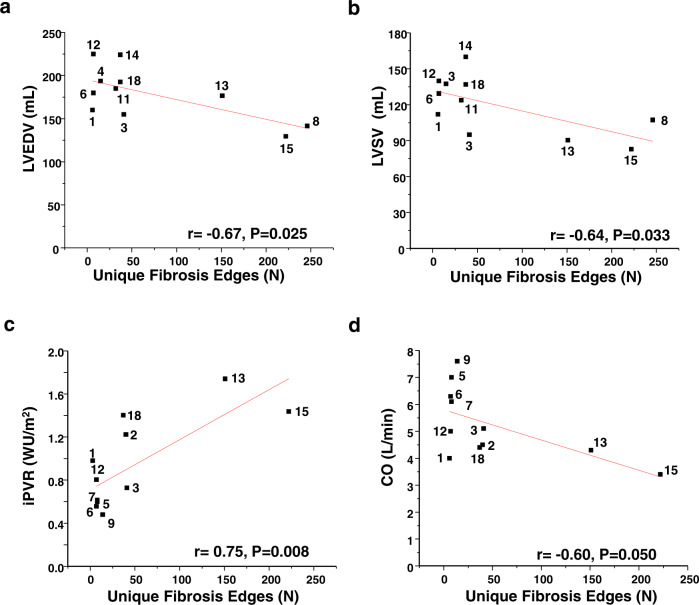


This has been corrected in both the PDF and HTML versions of the Article.

