# Author Correction: Individualized interactomes for network-based precision medicine in hypertrophic cardiomyopathy with implications for other clinical pathophenotypes

**DOI:** 10.1038/s41467-024-45814-x

**Published:** 2024-04-08

**Authors:** Bradley A. Maron, Rui-Sheng Wang, Sergei Shevtsov, Stavros G. Drakos, Elena Arons, Omar Wever-Pinzon, Gordon S. Huggins, Andriy O. Samokhin, William M. Oldham, Yasmine Aguib, Magdi H. Yacoub, Ethan J. Rowin, Barry J. Maron, Martin S. Maron, Joseph Loscalzo

**Affiliations:** 1 Division of Cardiovascular Medicine, Department of Medicine, Brigham and Women’s Hospital and Harvard Medical School, Boston, MA USA; 2 Channing Division of Network Medicine, Department of Medicine, Brigham and Women’s Hospital and Harvard Medical School, Boston, MA USA; 3 Division of Cardiovascular Medicine, University of Utah School of Medicine, Salt Lake City, UT USA; 4 Nora Eccles Harrison Cardiovascular Research and Training Institute (CVRTI), University of Utah School of Medicine, Salt Lake City, UT USA; 5 Hypertrophic Cardiomyopathy Center, Cardiology Division, Tufts Medical Center, Boston, MA USA; 6 Division of Pulmonary and Critical Care Medicine, Department of Medicine, Brigham and Women’s Hospital and Harvard Medical School, Boston, MA USA; 7 Department of Cardiac Surgery, Imperial College of London, London, UK; 8 The Magdi Yacoub Heart Center, Aswan, Egypt

**Keywords:** Network topology, Cardiac hypertrophy, Molecular medicine

Correction to: *Nature Communications* 10.1038/s41467-021-21146-y, published online 08 February 2021

The original version of this Article contained errors in Figure 2 and Figure 3. In figure 2, the network for HCM patient 4 was erroneously duplicated from HCM patient 3.

The incorrect version of Figure 2 is:


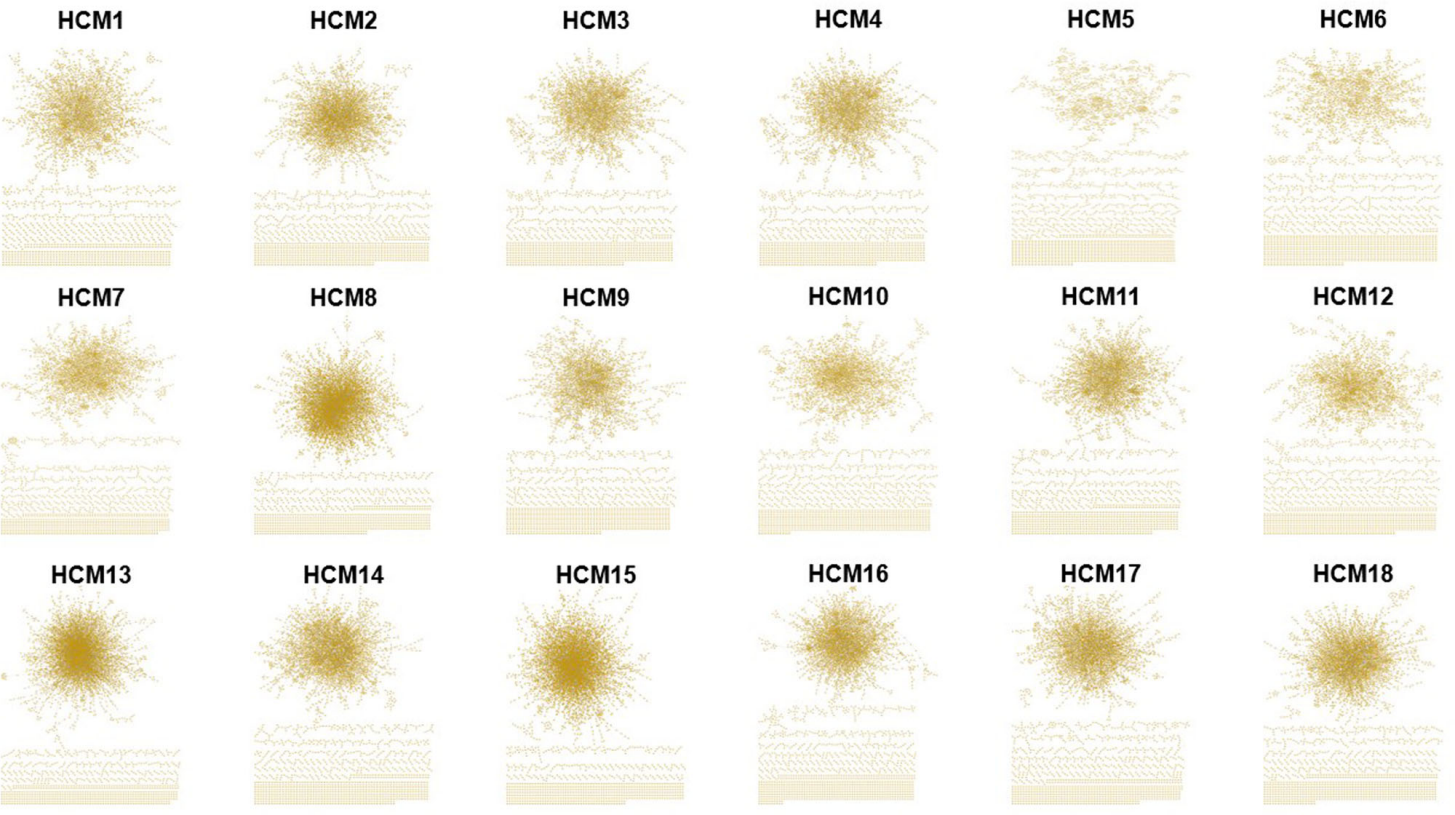


The correct version of Figure 2 is:
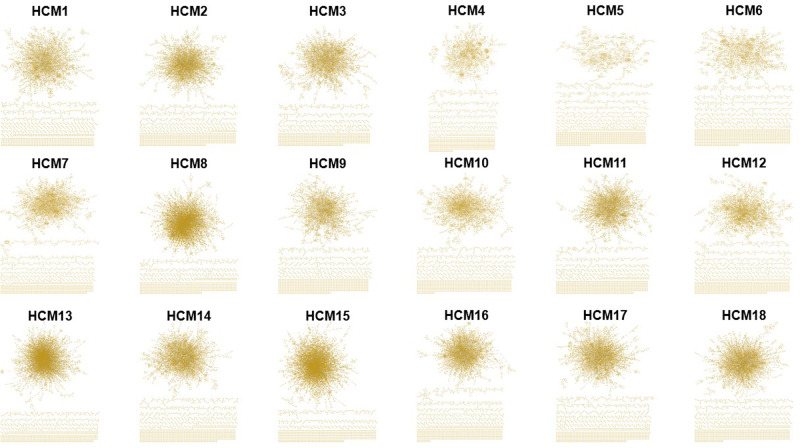


In Figure 3G, the HCM 4 data point was inadvertently omitted when transposing the linear regression curve inset from one file format to the finalized final format.

The incorrect version of Figure 3G is:


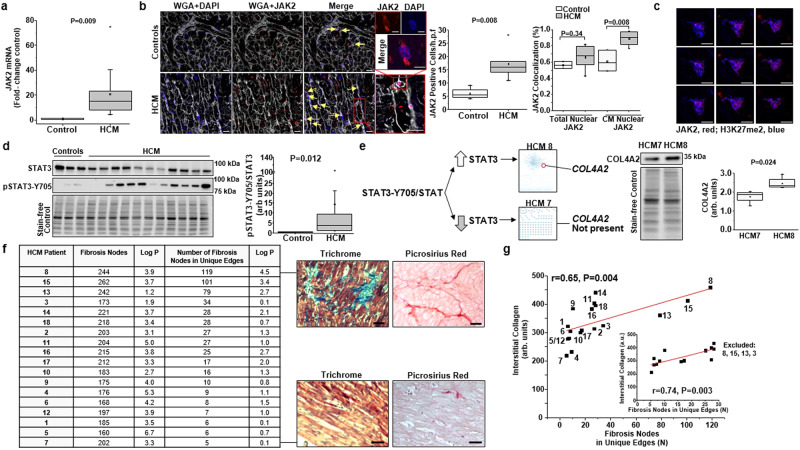


The correct version of Figure 3G is:


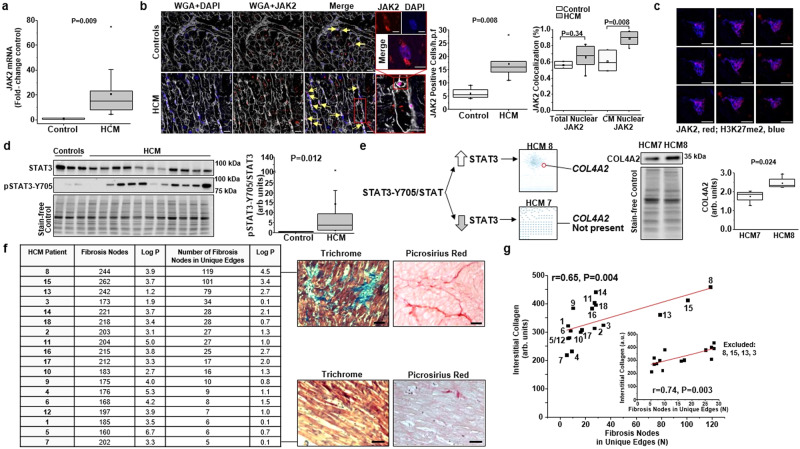


In addition, in the Supplementary Methods section of the Supplementary Information, the size of the consolidated human interactome was incorrectly reported. The incorrect sentence was ‘This new version of the consolidated human interactome has 15,849 proteins and 188,973 interactions and displays a scale-free topology (Supplementary Fig. 7).’. The correct sentence reads : ‘This new version of the consolidated human interactome has 15,489 proteins and 188,973 interactions and displays a scale-free topology (Supplementary Fig. 7).

These errors have been corrected in the HTML and PDF versions of the Article, and the HTML has been updated to include a corrected version of the Supplementary information.

