# Supplementary material for: Individualized interactomes for network-based precision medicine in hypertrophic cardiomyopathy with implications for other clinical pathophenotypes
Source: Nat Commun. 2021 Feb 8;12:873. doi: 10.1038/s41467-021-21146-y (PMC7870822; doi:10.1038/s41467-021-21146-y)
Supplement: Supplementary file 1 — Supplementary Information [file 41467_2021_21146_MOESM1_ESM.pdf]

March 21, 2024

Individualized Interactomes for Network-Based Precision Medicine in Hypertrophic Cardiomyopathy with Implications for Other Clinical Pathophenotypes

Supplementary Methods

Patient echocardiography. Transthoracic echocardiography was performed with measurements of left ventricular (LV) wall thickness and cavity dimensions, ejection fraction, and LV outflow tract gradient (estimated with continuous wave Doppler) obtained as previously reported.[1] Patients with LV outflow tract gradient of <50 mmHg at rest underwent symptom limited exercise (stress) echocardiography on a standard Bruce protocol to evaluate provocable outflow gradients.

Patient cardiac magnetic resonance imaging. Cardiac magnetic resonance imaging (CMR) studies were performed in 13 of the 18 HCM study patients (72%) using a 1.5 T clinical scanner with cine images obtained in 3 long-axis planes and sequential short axis slices with full LV coverage.[2] Late gadolinium enhancement (LGE) images to assess myocardial scar were acquired 10–15 min after intravenous administration of 0.2 mmol/kg gadolinium-DTPA using breath-held segmented inversion-recovery sequence. LGE quantification was performed by manually adjusting grayscale threshold to visually define areas of LGE, which were summed and expressed as proportion of total LV myocardial mass.

Patient cardiac catheterization. As part of clinical evaluation prior to myectomy, right heart catheterization was performed with the patient in the supine position at rest.[1] Most patients continued to take cardioactive medications administered for the purpose of controlling symptoms of heart failure in obstructive HCM. Right atrial, mean pulmonary artery pressure (mPAP), and pulmonary artery wedge pressure (PAWP) were measured at end-expiration averaged over ≥3 beats. Cardiac output (CO) was determined by the thermodilution method.[3] Pulmonary vascular resistance was calculated as: (mPAP-PAWP)/CO and indexed to body surface area (iPVR).

Tissue harvesting. Anterior septal tissue samples were acquired from HCM patients at the time of surgical septal myectomy (G.H.) and cut into ~200 mg aliquots. Tissue sections were snap frozen or stored in 4% paraformaldehyde for paraffin embedding. For controls, the LV free-wall tissue was collected in the operating room immediately after its removal from the operating surgeon, divided, and promptly processed by fixation in 10% buffered formalin or flash freezing in liquid nitrogen, then stored at -80° C for later molecular analysis (S.G.D.). Collectively, these samples were received and analyzed further at a single laboratory location (S.S., R-S.W., B.A.M., J.L.).

RNA-Seq analysis. For controls and HCM patients, frozen hearts (0.3 mg) were later incubated in RNAlater for 2 h at 4°C and tissue was disrupted using GentleMACS tissue dissociator (Miltenyi Biotec). RNA was isolated using the RNeasy Fibrous Tissue Mini Kit (Qiagen, #74704) per the manufacturer's instructions. The methods for analyzing and assembling RNA-Seq transcriptomic data were published previously,[4] but are restated here for completeness. Sequencing data were processed to remove any adaptor, PCR primers, and low-quality transcripts using FASTQC and FASTX. These high quality, clean reads were aligned against the human genome using tophat2 and bowtie2 packages (http://tophat.cbcb.umd.edu/). Gene expression measurement was performed from aligned reads by counting the unique reads. The genome version to which we aligned our sequencing reads is *Homo sapiens* (human) genome assembly GRCh37 (hg19). The transcriptome version that was used for the analysis is Homo_sapiens.GRCh37.75.cdna.all.fa.gz from Ensemble (https://uswest.ensembl.org/index.html). The read count-based gene expression data were normalized on the basis of library complexity and gene variation using the R package EdgeR. The normalized count data were compared among groups using a negative binomial model to identify differentially expressed genes. To validate data comparing HCM vs. DCM network

node and edge overlap, we used RNA-Seq data from the following Geo database entries: GSE36961 vs. GSE116250 and GSE130036 and GSE116250.

DNA Sequencing. Gene sequencing was performed on DNA isolated from HCM samples. GATK (Genome Analysis Toolkit)[5] was used to classify the variants into SNPs and Indels, and filtered out variants by allele frequency (0.5%) in the normal population, using data from the 1,000 Genomes Project[6] and ExAC (The Exome Aggregation Consortium).[7] Those SNPs that annotated as common in dbSNP138 were removed; additionally, SnpEff[8] was used to annotate the variants to include only variants with potentially deleterious effects, such as exon loss variants, frameshift variants, stop gained variants, stop lost variants, and missense variants. We used ANNOVAR,[9] which integrates SIFT and PolyPhen to predict the possible impact of an amino acid substitution on the structure/function of a protein, and removed those variants that do not have deleterious effects on protein function. Pathogenic and likely pathogenic variants were collected from multiple references and the Clinvar NCBI database.[10,11] Sequencing data of sufficient quality were available for 89% of the HCM cohort.

Quantitative real time PCR. The methods used in this study for isolating RNA and performing RT-qPCR have been reported previously.[4] Relative JAK2 (probe Hs0061050_m1 with Amplicon length 79 bp, Life Technologies) expression was calculated using the comparative cycle threshold method referenced to POLR2A (probe Hs00172187_m1, Life Technologies) mRNA. The JAK2 mRNA transcript quantities are expressed as fold-change compared to control LV.

Immunoblotting. Proteins from LV samples were size-fractionated electrophoretically using sodium dodecyl sulfate polyacrylamide gel (SDS-PAGE) and transferred to polyvinylidene

fluoride membranes according to methods reported previously.[12] The membranes were incubated with rabbit anti-human anti-STAT3 (Cell Signaling, #9145S), rabbit anti-human anti-STAT3-P-Y705 antibodies (Cell Signaling, #4904S), rabbit anti-JAK2 (Cell Signaling, #320L), and rabbit anti-COL4A2 (Abcam, #125208) antibodies at a dilution 1:1,000 overnight at 4°C. The membranes were incubated with peroxidase-labeled secondary antibody, and visualized using the ECL detection system. Densitometry was performed using the ChemiDoc Touch System (BioRad) and standardized to signal generated by stain-free gels, which served as loading controls.

Endophenotype enrichment analysis. We collected genes associated with 52 representative endophenotypes that might be relevant to HCM and also have more than 20 associated genes from Phenopedia.[13] Since hypoxia and oxidative stress are common endophenotypes in cardiovascular diseases, we decided to add them to our endophenotype list, as well. We compiled hypoxia-response genes from our in-house microarray gene expression data[14] and a public microarray dataset.[15] Oxidative stress genes were extracted from the Gene Ontology (GO) database (http://geneontology.org/) by using the GO term: Oxidative stress. We next used the hypergeometric test[16] to determine whether or not an individualized HCM network is significantly enriched with genes associated with these 54 endophenotypes compared to the human interactome as the background. We found 30 endophenotypes have P-values lower than 0.01 in at least one patient. A heatmap of $-\log_{10}P$ is shown in Supplementary Fig. 2a. In this heatmap, the rows correspond to significant endophenotypes, and the columns denote 18 patients. The heatmap is depicted as $-\log_{10}P$, where p is the P-value from the endophenotype enrichment analysis. The software matrix2png[16] was used to graph all heatmaps. A list of genes for each significant endophenotype is provided in Supplementary Data File 10.

Immunofluorescence in situ. LV samples were oriented in the long axis and short axis, and processed/embedded in paraffin using a Hypercenter XP System and Embedding Center (Shandon). The paraffin-embedded lung tissue was cut into 5-μm sections and stained with wheat germ agglutinin labelled with CF® dye (WGA) (Biotum, #29026-1) or a rabbit anti-human antibody against JAK2 (Cell signaling, #3230). Zen software (ZEISS Microscopy) was used to visualize images and quantify stain intensity including co-localization of two different immunofluorescence signals. For experiments focusing on imaging the cell nucleus in specifically, immunofluorescent images were taken using LSM 800 Airyscan microscope and processed in Superresolution (3D, Auto) Airyscan mode. The Z-stack images were acquired at 0.16 μm intervals for at least 2.4 μm. Data are expressed as the number of JAK2-positive cells per high powered field (200x magnification) or the percent of JAK2-nuclear colocalization for all DAPI-stained cells or DAPI-stained cardiomyocytes. The average quantity from 3-5 images per sample was considered as a single datapoint.

Cardiac histology. Paraffin-embedded LV samples from HCM and healthy control patients were cut in cross-section at a thickness of 5 μm. Sections were stained using the Picrosirius Red Stain Kit and Masson Trichrome Stain Kit Sigma according to manufacturer's instructions. Images obtained at 200x magnification were visualized under non-polarized light and imaged using an LSM 700 Flexible Confocal Microscope. Interstitial collagen was the focus of analyses in this study, and defined here as collagen intermingled with myocardial tissue excluding scars, endocardium, intramyocardial coronary vessels (>0.5 mm), and replacement scar, as published previously.[17] Photomicrographs were converted to RGB images and deconvoluted using ImageJ.[18] Focusing on the blue channel, the threshold setting was adjusted to the minimum required for

illuminating blue stain, and the signal intensity was quantified automatically. The average result for 3-5 images represented findings from a single patient sample.

Consolidated human interactome. To build a comprehensive human interactome, we compiled human physical molecular interaction data from different sources, including protein-protein interactions, protein complexes, kinase-substrate interactions, and signaling pathways. Protein-protein interactions from several high-throughput yeast-two-hybrid studies as well as the high-quality protein-protein interactions from the literature were compiled from the CCSB Human Interactome.[19-23] We also collected binary protein-protein interactions from other laboratories.[24,25] A protein complex is a group of two or more associated polypeptide chains linked by non-covalent protein-protein interactions. Protein-protein co-complex interactions were compiled from different high-profile publications.[26-32] In addition, we also incorporated experimental signaling interactions and kinase-substrate interactions as well as high-quality literature-based signaling interactions as they are involved in various biological pathways.[33-36] This new version of the consolidated human interactome has 15,489 proteins and 188,973 interactions and displays a scale-free topology (Supplementary Fig. 7).

Topological features of individual HCM networks. We used NetworkAnalyzer in Cytoscape to analyze the topological features of individual HCM networks.[37] In addition to network size (number of nodes and number of edges), we consider seven other topological features, including clustering coefficients, network density, network diameter, network heterogeneity, network centralization, characteristic path length, and average number of neighbors. Clustering coefficient is a measure of the degree to which nodes in a network tend to cluster together and quantifies the abundance of connected triangles. Network density describes the portion of the potential edges in

a network that are actual edges. Network diameter is the shortest distance between the two most distant nodes in a network. Network heterogeneity is defined as the variance of node degrees in a network. Centrality identifies the most important vertices within a network, and network centralization characterizes how much variation is there in the centrality scores among the nodes. Characteristic path length is defined as the average of the shortest path length over all nodes in a network. The average number of neighbors is the average degree (number of connections) of all nodes in a network. Collectively, these measures characterize different topological features of a large network.

HCM		Controls	
Parameter (N=18)		Parameter (N=5)	
Males (%)	11 (61)	Males (%)	1 (20)
Age	50.1 ± 13	Age	45 ± 7.6
Family history HCM (%)	4 (22)	Co-morbidities (N=5)	
FH HCM sudden cardiac death (%)	2 (11)	Hypertension (%)	1 (20)
NSVT on Holter monitor	7 (39)	Substance abuse (%)	4 (80)
Syncope	5 (28)	Stroke/PFO closure (%)	1 (20)
Echocardiographic characteristics (N=18)		None (%)	1 (20)
Max LV thickness (mm)	20 [18-22]	Cause of death	
LA size (mm)	43.7 ± 6.6	Patient	
LV EF (%)	65 [65-65]	1	Head trauma/Blunt injury/MVA
Resting LVOT gradient (mmHg)	25 [0-80]	2	Intracranial bleeding
Peak exercise LVOT gradient (mmHg)	69.5 ± 52.9	3	Head trauma/Blunt injury
Pre-myectomy NYHA FC		4	Anoxia/Drug intoxication
II (%)	5 (28)	5	Head trauma/Blunt injury/Accident Non-MVA
III (%)	13 (72)		
Cardiac MRI characteristics (N=13)			
LGE positivity (%)	8 (62)		
LGE %	1.1 [0-5.1]		
LV EDV (mL)*	178 ± 30.6		
LV ESV (mL)*	59 ± 16		
LV SV (mL)*	120 ± 24		
LV mass (g)*	147 ± 44		
RV EDV (mL)*	129 ± 30		
RV ESV (mL)*	53 ± 13		
RV SV (mL)*	76 ± 23		
Right heart catheterization characteristics (N=12)			
RA (mmHg)	8.5 ± 4.6		
mPAP (mmHg)	25 ± 8.8		
PAWP (mmHg)	15 ± 6.7		
Cardiac output (L/min)	5.2 ±1.2		
Indexed PVR (Wood units/m^2)	0.8 [0.6-1.4]		

Supplementary Table 1. Demographic and clinical characteristics of the HCM and control populations. HCM, hypertrophic cardiomyopathy; FH, family history; NSVT, non-sustained ventricular tachycardia; LV, left ventricle, LA, left atrial; EF, ejection fraction; LVOT, left ventricular outflow tract; NYHA FC, New York Heart Association Functional Class; LGE, late gadolinium enhancement; EDV, end-diastolic volume; ESV, end-systolic volume; SV, stroke volume; RA, right atrial; mPAP, mean pulmonary artery pressure; PAWP, pulmonary artery wedge pressure; PVR, pulmonary vascular resistance. Categorical data are expressed as N (%), continuous data are expressed

as mean ± SD, and non-continuous data are expressed as median [IQR].*N=11. Individual data points are provided in Supplementary Data File 1.

	HCM 2	HCM 3	HCM 4	HCM 5	HCM 6	HCM 7	HCM 8	HCM 9	HCM 10	HCM 11	HCM 12	HCM 13	HCM 14	HCM 15	HCM 16	HCM 17	HCM 18
HCM 1	0.85	0.84	0.88	0.84	0.84	0.85	0.82	0.86	0.86	0.85	0.85	0.87	0.85	0.84	0.83	0.85	0.84
HCM 2		0.99	0.50	0.99	0.99	0.99	0.97	0.98	1.00	1.00	0.98	0.98	0.98	0.91	0.98	0.98	0.99
HCM 3			0.50	0.99	1.00	1.00	0.97	0.98	1.00	1.00	0.98	0.99	0.99	0.89	0.99	0.97	1.00
HCM 4				0.50	0.50	0.52	0.49	0.54	0.51	0.51	0.53	0.55	0.53	0.56	0.49	0.52	0.50
HCM 5					0.99	1.00	0.99	0.99	0.99	0.99	0.99	0.99	0.99	0.92	0.99	0.99	0.99
HCM 6						1.00	0.97	0.98	1.00	1.00	0.98	0.98	0.99	0.89	0.99	0.97	1.00
HCM 7							0.98	0.99	1.00	1.00	0.99	0.99	0.99	0.91	0.99	0.98	1.00
HCM 8								0.97	0.98	0.97	0.98	0.97	0.98	0.94	0.98	0.97	0.97
HCM 9									0.99	0.98	1.00	0.99	1.00	0.95	0.99	1.00	0.98
HCM 10										1.00	0.99	0.99	0.99	0.91	0.99	0.98	1.00
HCM 11											0.98	0.99	0.99	0.89	0.99	0.97	1.00
HCM 12												0.99	1.00	0.96	0.98	1.00	0.98
HCM 13													0.99	0.93	0.99	0.99	0.98
HCM 14														0.94	0.99	0.99	0.99
HCM 15															0.91	0.96	0.89
HCM 16																0.98	0.99
HCM 17																	0.97

Supplementary Table 2. Gene replicate quality from RNA-Seq analysis for hypertrophic cardiomyopathy (HCM) patients. The transcriptomic profile of anterior septal myectomy specimens from patients with hypertrophic cardiomyopathy (HCM) was analyzed (N=18). The Pearson correlation coefficient for replicates from all transcriptomic features is provided as a comparison between patient pairs.

	Control 2	Control 3	Control 4	Control 5
Control 1	0.98	0.97	0.97	0.98
Control 2		0.97	0.95	0.99
Control 3			0.95	0.96
Control 4				0.93

Supplementary Table 3. Gene replicate quality from RNA-Seq analysis for healthy controls patients. The transcriptomic profile of left ventricular myocardial samples from rejected heart transplant donors serving as healthy controls was analyzed. The Pearson correlation coefficient for replicates from all transcriptomic features is provided as a comparison between control pairs.

	Network Characteristic								
	No. nodes	No. edges	Clustering coefficient	Density	Diameter	Heterogeneity	Centralization	Characteristic path length	Ave. No. of neighbors
HCM1	3328	3244	0.006	0.00059	23	1.161	0.012	8.409	1.95
HCM2	3767	4043	0.007	0.00057	20	1.215	0.012	7.403	2.15
HCM3	3355	3443	0.005	0.00061	30	1.035	0.010	8.861	2.05
HCM4	2928	2645	0.004	0.00062	25	1.324	0.016	9.079	1.81
HCM5	2474	2014	0.004	0.00066	30	1.039	0.014	11.135	1.63
HCM6	2891	2531	0.006	0.00061	22	1.074	0.013	9.439	1.75
HCM7	3727	3726	0.004	0.00054	22	1.200	0.012	8.364	1.99
HCM8	4980	6626	0.009	0.00053	20	1.263	0.013	6.686	2.66
HCM9	3056	2926	0.006	0.00063	22	1.168	0.014	8.354	1.92
HCM10	3421	3308	0.003	0.00057	32	1.063	0.009	8.727	1.93
HCM11	3531	3666	0.006	0.00059	25	1.206	0.012	7.822	2.08
HCM12	3525	3393	0.005	0.00055	24	1.272	0.013	8.593	1.93
HCM13	5031	6470	0.008	0.00051	23	1.289	0.015	6.825	2.57
HCM14	4062	4334	0.007	0.00053	21	1.276	0.014	7.594	2.13
HCM15	4937	6479	0.008	0.00053	22	1.304	0.011	6.700	2.63
HCM16	3925	4112	0.006	0.00053	29	1.290	0.011	7.611	2.10
HCM17	3938	4163	0.005	0.00054	20	1.240	0.011	7.681	2.11
HCM18	4051	4369	0.005	0.00053	23	1.109	0.007	7.836	2.16
DCM1	3973	3889	0.006	0.00049	27	1.12	0.008	9.081	1.963
DCM2	3851	3824	0.005	0.00052	25	1.132	0.01	8.65	1.986
DCM3	4470	4568	0.009	0.00046	26	1.064	0.009	8.961	2.044
DCM4	5687	6887	0.005	0.00043	24	1.097	0.008	8.048	2.422
DCM5	4745	5193	0.007	0.00046	29	1.149	0.013	8.587	2.189
DCM6	5332	6261	0.008	0.00044	25	1.156	0.012	7.993	2.348
P-Value	0.010	0.083	0.252	0.000	0.238	0.073	0.045	0.421	0.568

Supplementary Table 4. Characteristics of each HCM and DCM patient-specific network. HCM, hypertrophic cardiomyopathy; DCM, dilated cardiomyopathy; Ave., average; No. number. The P-Values reflect analyses comparing differences between the HCM cohort vs. DCM cohort for each network parameter.

HCM Patient Network	Network: Original Filter		Network: Revised Filter		Similarity Between Unfiltered and Filtered Networks (%)	
	Proteins	PPIs	Proteins	PPIs	Proteins	PPIs
HCM1	3328	3244	2916	2893	97.1	96.3
HCM2	3767	4043	3407	3706	97.1	96.0
HCM3	3355	3443	3065	3194	97.4	96.5
HCM4	2928	2645	2410	2170	97.4	96.3
HCM5	2474	2014	2106	1691	96.9	96.4
HCM6	2897	2531	2516	2199	96.9	95.9
HCM7	3727	3726	3173	3163	97.7	96.6
HCM8	4980	6626	4311	5747	97.8	96.8
HCM9	3056	2926	2693	2608	96.9	96.3
HCM10	3421	3308	3058	3001	97.6	96.3
HCM11	3531	3666	3163	3346	97.1	96.2
HCM12	3525	3393	2928	2789	97.3	96.3
HCM13	5031	6470	4444	5762	97.2	96.5
HCM14	4062	4334	3566	3818	97.0	96.1
HCM15	4937	6479	4266	5657	97.8	96.7
HCM16	3925	4112	3313	3492	97.6	96.3
HCM17	3938	4163	3362	3541	97.4	96.6
HCM18	4051	4369	3626	3954	96.9	95.7

Supplementary Table 5. Similarity in hypertrophic cardiomyopathy patient-specific networks. Differences in protein and protein-protein interactions (PPIs) in HCM patient-specific networks are presented, as determined by analyzing the RNA-Seq dataset with the original filtering method (inclusive of genes with ≥10 counts in ≥1 sample[s] from the healthy cohort) or a revised filtering method (inclusive of genes with ≥10 read counts in all samples from the healthy cohort).

			DCM Patient											
			Node overlap						Edge Overlap					
DCM Patient	Network size (nodes)	Network size (edges)	1	2	3	4	5	6	1	2	3	4	5	6
1	3,973	3,899	1	0.68	0.69	0.79	0.72	0.77	1	0.46	0.38	0.43	0.42	0.44
2	3,851	3,824		1	0.68	0.79	0.72	0.78		1	0.37	0.44	0.44	0.46
3	4,470	4,568			1	0.77	0.68	0.74			1	0.42	0.37	0.41
4	5,687	6,887				1	0.79	0.79				1	0.51	0.51
5	4,745	5,193					1	0.78					1	0.52
6	5,332	6,261						1						1
Mean			0.73	0.73	0.71	0.79	0.74	0.77	0.43	0.43	0.39	0.46	0.45	0.47
SD			0.05	0.04	0.01	0.05	0.02	0.03	0.04	0.02	0.04	0.06	0.05	0.05

Supplementary Table 6. Size and feature overlap across individual patient networks for the dilated cardiomyopathy (DCM) cohort. The transcriptomic profile of patients with dilated cardiomyopathy (DCM)[38] (N=6) was analyzed using the same analytical method to develop individualized networks for hypertrophic cardiomyopathy patients (see Fig. 1 for details). Nodes (representing proteins in the networks) and edges (representing links or physical associations between proteins in the networks) common to individual-patient DCM network pairs are presented. 1, full overlap; 0, no overlap. 1, full overlap; 0, no overlap. SD, standard deviation.

	DCM1	DCM2	DCM3	DCM4	DCM5	DCM6
HCM1	3.28E-12	<1.0E-16	1.26E-11	2.42E-13	<1.0E-16	<1.0E-16
HCM2	3.28E-12	<1.0E-16	9.95E-12	<1.0E-16	<1.0E-16	<1.0E-16
HCM3	<1.0E-16	<1.0E-16	<1.0E-16	<1.0E-16	<1.0E-16	<1.0E-16
HCM4	<1.0E-16	<1.0E-16	4.92E-12	<1.0E-16	<1.0E-16	<1.0E-16
HCM5	<1.0E-16	<1.0E-16	5.18E-12	<1.0E-16	<1.0E-16	<1.0E-16
HCM6	<1.0E-16	<1.0E-16	<1.0E-16	<1.0E-16	<1.0E-16	<1.0E-16
HCM7	<1.0E-16	<1.0E-16	<1.0E-16	<1.0E-16	<1.0E-16	<1.0E-16
HCM8	<1.0E-16	<1.0E-16	2.64E-12	<1.0E-16	<1.0E-16	<1.0E-16
HCM9	5.87E-12	<1.0E-16	1.37E-11	3.28E-12	<1.0E-16	2.79E-14
HCM10	<1.0E-16	<1.0E-16	2.64E-12	<1.0E-16	<1.0E-16	<1.0E-16
HCM11	<1.0E-16	<1.0E-16	5.31E-12	<1.0E-16	<1.0E-16	<1.0E-16
HCM12	<1.0E-16	<1.0E-16	4.94E-12	<1.0E-16	<1.0E-16	<1.0E-16
HCM13	<1.0E-16	<1.0E-16	8.37E-13	<1.0E-16	<1.0E-16	<1.0E-16
HCM14	1.09E-11	<1.0E-16	2.16E-11	1.61E-12	<1.0E-16	4.90E-12
HCM15	<1.0E-16	<1.0E-16	6.27E-12	<1.0E-16	<1.0E-16	<1.0E-16
HCM16	1.55E-11	4.92E-12	2.26E-11	1.03E-11	9.43E-13	1.22E-11
HCM17	1.31E-12	<1.0E-16	1.07E-11	<1.0E-16	<1.0E-16	<1.0E-16
HCM18	<1.0E-16	<1.0E-16	6.06E-12	<1.0E-16	<1.0E-16	<1.0E-16

Supplementary Table 7. The P-Values for node overlap between personalized networks for patients with hypertrophic cardiomyopathy (HCM) and dilated cardiomyopathy (DCM). The significance of node overlap was assessed using the hypergeometric test in a space of 15,489 proteins and the P-values were adjusted by the Benjamini-Hochberg procedure for multiple comparison

	DCM1	DCM2	DCM3	DCM4	DCM5	DCM6
HCM1	0.05	0.11	0.32	0.05	0.06	0.12
HCM2	0.07	0.09	0.28	0.20	0.04	0.05
HCM3	0.50	0.17	0.59	0.15	0.19	0.16
HCM4	0.34	0.41	0.80	0.37	0.07	0.84
HCM5	0.11	0.12	0.23	0.11	0.23	0.21
HCM6	0.11	0.11	0.78	0.01	0.01	0.24
HCM7	0.19	0.11	0.32	0.15	0.10	0.39
HCM8	0.01	0.01	0.01	0.03	0.01	0.09
HCM9	0.03	0.01	0.22	0.07	0.01	0.04
HCM10	0.07	0.12	0.14	0.05	0.01	0.07
HCM11	0.15	0.17	0.57	0.29	0.25	0.66
HCM12	0.25	0.17	0.35	0.14	0.05	0.35
HCM13	0.11	0.21	0.16	0.12	0.05	0.28
HCM14	0.21	0.29	0.30	0.21	0.15	0.37
HCM15	0.16	0.27	0.29	0.21	0.03	0.17
HCM16	0.41	0.29	0.21	0.02	0.11	0.13
HCM17	0.32	0.23	0.21	0.11	0.02	0.25
HCM18	0.11	0.09	0.28	0.23	0.28	0.50

Supplementary Table 8. The P-Values for edge overlap using between personalized networks for patients with hypertrophic cardiomyopathy (HCM) and dilated cardiomyopathy (DCM). Edge overlap was assessed by the hypergeometric test in a space of 188,973 interactions; therefore, very small overlap between components from two networks could achieve statistical significance. The P-values were adjusted by the Benjamini-Hochberg procedure for multiple comparison.

HCM Patient	Gene Variant	Classification
7	MYBPC3: c.1928-2A>G	Pathogenic
8	MYBPC3: c.G2497A:p.A833T	Pathogenic
5	MYL2: c.C141A:p.N47K	Pathogenic
12	MYL2: c.G2429A:p.R810H	Likely Pathogenic

Supplementary Table 9. Putative HCM-causing gene variants in the study cohort patients.

HCM, hypertrophic cardiomyopathy.

HCM Patients #	Network size (nodes)	Network size (edges)	Fibrosis nodes	Fibrosis P-Value	Hypertrophy nodes	Hypertrophy P-Value
1	3,328	3,244	185	0.001318	65	0.435482
2	3,767	4,043	203	0.002924	78	0.230476
3	3,355	3,443	173	0.034445	67	0.361266
4	2,928	2,645	176	3.54E-05	58	0.408305
5	2,474	2,014	160	1.78E-06	49	0.426204
6	2,891	2,531	168	0.000351	57	0.423704
7	3,727	3,726	202	0.002283	82	0.092381
8	4,980	6,626	244	0.000711	94	0.097785
9	3,056	2,926	175	0.000491	64	0.241087
10	3,421	3,308	183	0.007395	65	0.517365
11	3,531	3,666	204	6.44E-05	77	0.117970
12	3,525	3,393	197	0.000606	78	0.092965
13	5,031	6,470	242	0.118740	101	0.270808
14	4,062	4,334	221	0.000962	88	0.103374
15	4,937	6,479	262	0.000944	90	0.658090
16	3,925	4,112	215	0.000842	79	0.310002
17	3,938	4,163	212	0.002283	83	0.171545
18	4,051	4,369	218	0.001915	82	0.285160

Supplementary Table 10. Description of patient-specific networks. The transcriptomic profile of anterior septal myectomy specimens from patients with hypertrophic cardiomyopathy (HCM) (N=18) was analyzed using a two-step method that included a correlation matrix and protein-protein interaction analysis resulting in individualized patient HCM networks. The number of nodes (representing proteins) and edges (representing links between nodes) in each network are provided. We also present the number of fibrosis nodes and hypertrophy nodes determined by gene ontology analyses. Enrichment of each network for fibrosis nodes and hypertrophy nodes was calculated by the hypergeometric test. The P-Values were adjusted by the Benjamini-Hochberg procedure for multiple comparison.

Fibrosis nodes common to all HCM patient networks	DE fibrosis nodes between HCM vs. Controls	LogFC	P-Value	FDR
A2M	JAK2	-1.13	2.2e-05	0.0014
ACTN4	IGF1R	-0.85	7.42e-07	0.0001
AKT1	HIF-1α	+0.89	9.91e-06	0.0008
APOE	JAK1	+0.70	0.0001	0.0044
BRCA1				
CHUK				
CLTC				
CSK				
CTNNB1				
GNAS				
GSK3B				
HIF1A				
HSPA1L				
IGF1R				
IKBKB				
IL6ST				
JAK1				
JAK2				
LMNA				
MAPK1				
MAPK11				
MAPK14				
MAPK9				
MDM2				
NFKBIA				
PAK1				
PLCG1				
PPP2R1A				
PTK2B				
RAC1				
REL				
RHOA				
ROCK1				
STAT1				
STAT3				
TP53				
TRAF6				
VIM				
XRCC1				
XRCC3				
YES1				

Supplementary Table 11. Fibrosis pathway nodes common to all individual HCM patient networks. The transcriptomic profile of anterior septal myectomy specimens from patients with hypertrophic cardiomyopathy (HCM) (N=18) was analyzed using a two-step method that included a correlation matrix and protein-protein interaction analysis resulting in individualize patient networks. Nodes (representing proteins) associated with fibrosis from a gene ontology analysis

that were common to all HCM networks are presented. The genes from this group that were also differentially expressed ($P<0.05$, $FDR<0.05$) between the HCM vs. control cohort ($N=5$) are presented. The P-values were generated using EdgeR, which implements a generalized linear model and provides false discovery rate (FDR) for multiple comparison. DE, differentially expressed; LogFC, log fold-change.

	Nodes involved in unique network edges	Unique network edges
HCM 1	180	107
HCM 2	438	300
HCM 3	832	617
HCM 4	120	70
HCM 5	89	49
HCM 6	83	42
HCM 7	135	78
HCM 8	1,880	1,722
HCM 9	154	101
HCM 10	234	133
HCM 11	474	325
HCM 12	92	50
HCM 13	1,294	974
HCM 14	1,387	249
HCM 15	1,648	1,472
HCM 16	300	191
HCM 17	205	120
HCM 18	539	334

Supplementary Table 12. Characteristics of unique network features for the HCM cohort. The transcriptomic profile of anterior septal myectomy specimens from patients with hypertrophic cardiomyopathy (HCM) (N=18) was analyzed using a two-step method that included a correlation matrix and protein-protein interaction analysis resulting in individualized patient networks. The number of nodes (representing proteins) and the number of edges (representing links between proteins) in the networks unique to any single patient are presented.

Supplementary References

1. Covella, M., et al. Mechanism of progressive heart failure and significance of pulmonary hypertension in obstructive hypertrophic cardiomyopathy. *Circ. Heart Fai.l* **10**, e003689 (2017).
2. Chan, R.H., et al. Prognostic value of quantitative contrast-enhanced cardiovascular magnetic resonance for the evaluation of sudden death risk in patients with hypertrophic cardiomyopathy. *Circulation* **130**, 484-95 (2014).
3. Opotowsky, A.R., et al. Thermodilution versus estimated Fick cardiac output measurement in clinical practice. *JAMA Cardiol.* **2**, 090-1099 (2017).
4. Samokhin, A.O., et al. NEDD9 targets *COL3A1* to promote endothelial fibrosis and pulmonary arterial hypertension. *Sci. Transl. Med.* **10**, 445B (2018).
5. McKenna, A.L., et al. The Genome Analysis Toolkit: a MapReduce framework for analyzing next-generation DNA sequencing data. *Genome Res.* **20**, 1297-303 (2010).
6. 1000 Genomes Project Consortium, Auton, A., et al. A global reference for human genetic variation. *Nature* **526**, 68-74 (2015).
7. Lek, M., Karczewski, K.J., & Minikel, E.V. Analysis of protein-coding genetic variation in 60,706 humans. *Nature* **536**, 285-91 (2016).
8. Cingolani, P.L., et al. A program for annotating and predicting the effects of single nucleotide polymorphisms, SnpEff: SNPs in the genome of Drosophila melanogaster strain w1118; iso-2; iso-3. *Fly. (Austin)* **6**, 80-92 (2012).
9. Wang, K., Li, M., & Hakonarson, H. ANNOVAR: functional annotation of genetic variants from high-throughput sequencing data. *Nucleic Acids Res.* **38,** e164 (2010).
10. Manrai, A.K., et al. Genetic misdiagnosis and the potential for health disparities. *N. Engl. J. Med.* **375**, 655-65 (2016).
11. Maron, B.J., Maron, M.S. & Semsarian, C. Genetics of hypertrophic cardiomyopathy after 20 years: clinical perspectives. *J. Am. Coll. Cardiol.* **60**, 705-15 (2012).

12. Maron, B.A., et al. Aldosterone inactivates the endothelin-B receptor via a cysteinyl thiol redox switch to decrease pulmonary endothelial nitric oxide levels and modulate pulmonary arterial hypertension. *Circulation* **126**, 963-974 (2012).

13. Yu, W., Clyne, M.,. Khoury, M.J., & Gwinn., M. Phenopedia and Genopedia: disease-centered and gene-centered views of the evolving knowledge of human genetic associations. *Bioinformatics* **26**,145–146 (2010).

14. Wang, R.S., Oldham, W.M., & Loscalzo, J. Network-based association of hypoxia-responsive genes with cardiovascular diseases. *New. J. Phys.* **16**, 105014 (2014)

15. Manalo, D.J., et al. Transcriptional regulation of vascular endothelial cell responses to hypoxia by HIF-1. *Blood* **105**, 659-69 (2005)

16. Spiegel, M. R. Theory and Problems of Probability and Statistics. New York: McGraw-Hill, pp. 113-114, 1992.

17. Moravsky, G., et al. Myocardial fibrosis in hypertrophic cardiomyopathy. *JACC: Cardiovasc. Imag.* **6**, 587-596 (2013).

18. Chen, Y., Yu, Q., & Cang-Bao, X. A convenient method for quantifying collagen fibers in atherosclerotic lesions by ImageJ softward. *Int. J. Clin. Exp. Med.* **10**, 14904-14-10 (2017).

19. Rual, J.,F., et al. Towards a proteome-scale map of the human protein-protein interaction network. *Nature* **437**,1173-8 (2005).

20. Venkatesan, K. et al. An empirical framework for binary interactome mapping. *Nat. Methods* **6**, 83-90 (2009).

21. Yu, H. et al. Next-generation sequencing to generate interactome datasets. *Nat. Methods* **8**, 478-80 (2011).

22. Rolland, T. et al. A proteome-scale map of the human interactome network. *Cell* **159**, 1212-1226 (2014).

23. Yang, X. et al. Widespread expansion of protein interaction capabilities by alternative splicing. *Cell* **164**, 805-17 (2016).

24. Stelzl, U. et al. A human protein-protein interaction network: a resource for annotating the proteome. *Cell* **122**, 957-68 (2005).

25. Yachie, N. et al. Pooled-matrix protein interaction screens using Barcode Fusion Genetics. *Mol. Syst. Biol.* **12,** 863 (2016).

26. Ewing, R.,M., et al. Large-scale mapping of human protein-protein interactions by mass spectrometry. *Mol. Syst. Biol.* **3**, 89 (2007).

27. Havugimana, P.C. et al., A census of human soluble protein complexes. *Cell* **150**, 1068-81 (2012).

28. Kristensen, A.R., Gsponer, J., & Foster, L.J. A high-throughput approach for measuring temporal changes in the interactome. *Nat. Methods* **9**, 907-9 (2012).

29. Huttlin, E.L. et al., The BioPlex network: a systematic exploration of the human interactome. *Cell* **162**, 425-440 (2015).

30. Wan, C. Panorama of ancient metazoan macromolecular complexes. *Nature* **525**, 339-44 (2015).

31. Hein, M.Y., et al. A human interactome in three quantitative dimensions organized by stoichiometries and abundances. *Cell* **163**, 712-23 (2015).

32. Huttlin, E.L., et al. Architecture of the human interactome defines protein communities and disease networks. *Nature* **545**, 505-509 (2017).

33. Vinayagam, A., et al. A directed protein interaction network for investigating intracellular signal transduction. *Sci Signal* **4**, rs8 (2011).

34. Türei D, Korcsmáros T, & Saez-Rodriguez J. OmniPath: guidelines and gateway for literature-curated signaling pathway resources. *Nat. Methods* **13**, 966-967 (2016).

35. Hornbeck, P.V., et al. PhosphoSitePlus, 2014: mutations, PTMs and recalibrations. *Nucleic Acids Res.* **43**(Database issue), D512-20 (2015).

36. Newman, R.H., et al. Construction of human activity-based phosphorylation networks. *Mol. Syst. Biol.* **9**, 655 (2013).

37. Assenov, Y., Ramírez, F., Schelhorn, S.E., Lengauer, T., & Albrecht, M. Computing topological parameters of biological networks. *Bioinformatics* **24**, 282-4 (2008).
