# Supplementary material for: Individualized interactomes for network-based precision medicine in hypertrophic cardiomyopathy with implications for other clinical pathophenotypes
Source: Nat Commun. 2021 Feb 8;12:873. doi: 10.1038/s41467-021-21146-y (PMC7870822; doi:10.1038/s41467-021-21146-y)
Supplement: Supplementary file 2 — Description of Additional Supplementary Files [file 41467_2021_21146_MOESM2_ESM.pdf]

Description of Additional Supplementary Files

File Name: Supplementary Data 1
Description: **Data metafile for each hypertrophic cardiomyopathy patient and healthy control.** NSVT, non-sustained ventricular tachycardia; Max, maximum; LV, left ventricle; EF, ejection fraction; LVOT, left ventricular outflow tract; NYHA FC, New York Heart Association Functional Class; LGE, late gadolinium enhancement; EDV, end-diastolic volume; ESV, end-systolic volume; SV, stroke volume; RA, right atrial; mPAP, mean pulmonary artery pressure; PAWP, pulmonary artery wedge pressure; iPVR, indexed pulmonary vascular resistance.

File Name: Supplementary Data 2
Description: **Overlap in network proteins between patient-specific hypertrophic cardiomyopathy (HCM) networks from our cohort and DCM networks derived from the GEO databases GSE116250, respectively.** Additionally, mean network node overlap between patients with within the DCM cohort is provided.

File Name: Supplementary Data 3
Description: **Overlap in network edges between patient-specific hypertrophic cardiomyopathy (HCM) networks from our cohort and DCM networks derived from the GEO databases GSE116250, respectively.** Additionally, mean network edge between patients with within the HCM cohort is provided.

File Name: Supplementary Data 4
Description: **Overlap in proteins between patient-specific hypertrophic cardiomyopathy (HCM) and dilated cardiomyopathy (DCM) networks derived from the GEO databases GSE36961 and GSE116250, respectively.** The overlap in network nodes between HCM and DCM is 0.24 ± 0.04. Additionally, mean network node overlap between patients within the HCM cohort is provided.

File Name: Supplementary Data 5
Description: **Overlap in edges between patient-specific hypertrophic cardiomyopathy (HCM) and dilated cardiomyopathy (DCM) networks derived from the GEO databases GSE36961 and GSE116250, respectively.** The overlap in network edges between HCM and DCM is 0.01 ± 0.01. Additionally, mean network edge overlap between patients within the HCM cohort is provided.

File Name: Supplementary Data 6
Description: **Overlap in proteins between patient-specific hypertrophic cardiomyopathy (HCM) and dilated cardiomyopathy (DCM) networks derived from the GEO databases GSE130036 and GSE116250, respectively.** The overlap in network nodes between HCM and DCM is 0.23 ± 0.01. Additionally, mean network node overlap between patients within the HCM cohort is provided.

File Name: Supplementary Data 7
Description: **Overlap in edges between patient-specific hypertrophic cardiomyopathy (HCM) and dilated cardiomyopathy (DCM) networks derived from the GEO databases GSE130036 and GSE116250, respectively.** The overlap in network edges between HCM and DCM is 0.01 ± 0.00. Additionally, mean network edge overlap between patients within the HCM cohort is provided.

File Name: Supplementary Data 8
Description: **Quantitative data for endophenotypes enriched in the hypertrophic cardiomyopathy (HCM) cohort after adjusting for multiple comparisons.** Data were analysed using the one-tailed hypergeometric test and the P-values were adjusted by the Benjamini-Hochberg procedure for multiple comparison.

File Name: Supplementary Data 9
Description: **Quantitative data for endophenotypes enriched in the dilated cardiomyopathy (DCM) cohort after adjusting for multiple comparisons.** Data were analysed using the one-tailed hypergeometric test and the P-values were adjusted by the Benjamini-Hochberg procedure for multiple comparison.

File Name: Supplementary Data 10
Description: **Phenopedia gene list for each of significant HCM endophenotypes.**
