# Supplementary material for: Individualized interactomes for network-based precision medicine in hypertrophic cardiomyopathy with implications for other clinical pathophenotypes
Source: Nat Commun. 2021 Feb 8;12:873. doi: 10.1038/s41467-021-21146-y (PMC7870822; doi:10.1038/s41467-021-21146-y)
Supplement: Supplementary file 13 — Reporting Summary [file 41467_2021_21146_MOESM13_ESM.pdf]

nature research

Corresponding author(s): Bradley A. Maron, M.D.

Last updated by author(s): Jan 9, 2021

Reporting Summary

Nature Research wishes to improve the reproducibility of the work that we publish. This form provides structure for consistency and transparency in reporting. For further information on Nature Research policies, see our Editorial Policies and the Editorial Policy Checklist.

Statistics

For all statistical analyses, confirm that the following items are present in the figure legend, table legend, main text, or Methods section.

n/a	Confirmed	
☐	☒	The exact sample size (n) for each experimental group/condition, given as a discrete number and unit of measurement
☐	☒	A statement on whether measurements were taken from distinct samples or whether the same sample was measured repeatedly
☐	☒	The statistical test(s) used AND whether they are one- or two-sided<br>*Only common tests should be described solely by name; describe more complex techniques in the Methods section.*
☐	☒	A description of all covariates tested
☐	☒	A description of any assumptions or corrections, such as tests of normality and adjustment for multiple comparisons
☐	☒	A full description of the statistical parameters including central tendency (e.g. means) or other basic estimates (e.g. regression coefficient) AND variation (e.g. standard deviation) or associated estimates of uncertainty (e.g. confidence intervals)
☐	☒	For null hypothesis testing, the test statistic (e.g. F, t, r) with confidence intervals, effect sizes, degrees of freedom and P value noted<br>*Give P values as exact values whenever suitable.*
☒	☐	For Bayesian analysis, information on the choice of priors and Markov chain Monte Carlo settings
☒	☐	For hierarchical and complex designs, identification of the appropriate level for tests and full reporting of outcomes
☐	☒	Estimates of effect sizes (e.g. Cohen's d, Pearson's r), indicating how they were calculated

Our web collection on statistics for biologists contains articles on many of the points above.

Software and code

Policy information about availability of computer code

Data collection	The interactome was manually compiled from published large-scale studies on human protein-protein interactions. Genes associated with endophenotypes were manually collected from an open source Phenopedia. All the resources are referenced in the manuscript and available from the corresponding author upon request. No software was used for data collection.
Data analysis	Differentially expressed genes were identified using EdgeR which is a free Bioconductor software package. Network construction based on correlation changes was implemented by our own R and Python scripts. We have deposited our codes in a publicly available website (DOI: 10.5281/zenodo.4429826).

For manuscripts utilizing custom algorithms or software that are central to the research but not yet described in published literature, software must be made available to editors and reviewers. We strongly encourage code deposition in a community repository (e.g. GitHub). See the Nature Research guidelines for submitting code & software for further information.

Data

Policy information about availability of data

All manuscripts must include a data availability statement. This statement should provide the following information, where applicable:

- Accession codes, unique identifiers, or web links for publicly available datasets
- A list of figures that have associated raw data
- A description of any restrictions on data availability

The HCM RNA-seq data and HCM exome VCF files have been uploaded to the GEO database (accession ID: GSE160997). All of the source programming code used in this study has been uploaded to Gihub (https://github.com/bwh784/HCM), which is a platform for sharing software packages. Other data that support the findings of this study are available from the corresponding author upon reasonable request. The RNA-seq and DNA sequencing data that support the findings of this study

have been deposited in the GEO database. Accession ID GSE160997[https://www.ncbi.nlm.nih.gov/geo/query/acc.cgi?acc=GSE160997]. Other data that support the findings of this study are available from the corresponding author upon reasonable request.

Field-specific reporting

Please select the one below that is the best fit for your research. If you are not sure, read the appropriate sections before making your selection.

☒ Life sciences ☐ Behavioural & social sciences ☐ Ecological, evolutionary & environmental sciences

For a reference copy of the document with all sections, see nature.com/documents/nr-reporting-summary-flat.pdf

Life sciences study design

All studies must disclose on these points even when the disclosure is negative.

Sample size	The number of patient samples included in this study was chosen based on availability and budgetary constraints. Nonetheless, in view of a priori knowledge that hypertrophic cardiomyopathy (HCM) is a heterogeneous disease clinically (Maron BJ, et al. J Am Coll Cardiol 2014;64:83-99) and prior findings from multiplex datasets showing biological heterogeneity in HCM murine models (Vakrou S, et al. JCI Insight 2018;3(6):e94493), we anticipated that a minimum of 10 patients was required to permit the emergence of informative differences in patient-specific PPI networks.
Data exclusions	Upon optimizing the experimental methods, all data were included in the analyses unless a specific technical reason was present that confounded the interpretation of a finding. Specifically, technical problems that obscured clear visualization of data occurred for experiments relating to Figure 3A (real time qPCR experiments performed on myocardial tissue, which was due to primer optimization), Figure 3B (immunofluorescence on myocardial samples, due primarily to antibody concentration optimization), Figures 3D,E (western blot using homogenized myocardial samples, which was due primarily to problems with film development, antibody-target binding, or other typical methodological issues that may arise during this experimental protocol), and Figure 3F and Supplemental Figure 4D (trichrome staining, which was due primarily to problems related to staining intensity). If a technical limitation was not identified in an experiment, the data were included in the analysis presented in the manuscript.
Replication	For data in Figures 3A, 3B, 3D, 3E, 3F and Supplemental Figure 4D experimental data were reproduced across at least 3 iterations of the same experiment. For data in Figures 3A, 3B, 3D, and 3E, the experiments were performed on different days by author S.S.. Data presented in Figure 3F were replicated independently by author E.A. Data presented in Supplemental Figure 4 were performed by author B.A.M. In the absence of a technical problem with an experiment, data from replication experiments were always consistent with results from prior iterations of the experiments or included in the analyses as part of the data variance.
Randomization	Randomization was not relevant to this study, and could not have been achieved. The reason randomization was not relevant to this project is because there was no treatment intervention involved in this study, and there were no in vivo models used in this study.
Blinding	The senior authors (B.A.M., B.J.M.) were blinded to the patient condition for experiments presented in Figures 3B, 3D, 3E, 3F and Supplemental Figure 4. Senior author J.L. was blinded to the patient condition for experiments presented in Figures 3B, 3F and Supplemental Figure 4. The computational analyst who performed the network analysis and other in silico analyses (co-author R-S.W.) is not a trained biologist nor clinician. Therefore, she was not privy to the biological or clinical implications of computational results. This served as a form of blinding for all computational analyses, which informed the results in all of the remaining figures.

Reporting for specific materials, systems and methods

We require information from authors about some types of materials, experimental systems and methods used in many studies. Here, indicate whether each material, system or method listed is relevant to your study. If you are not sure if a list item applies to your research, read the appropriate section before selecting a response.

Materials & experimental systems

n/a	Involved in the study	
☐	☒	Antibodies
☒	☐	Eukaryotic cell lines
☒	☐	Palaeontology and archaeology
☒	☐	Animals and other organisms
☐	☒	Human research participants
☒	☐	Clinical data
☒	☐	Dual use research of concern

Methods

n/a	Involved in the study	
☒	☐	ChIP-seq
☒	☐	Flow cytometry
☒	☐	MRI-based neuroimaging

Antibodies

Antibodies used	Western Blots:<br>1. Anti-STAT3 (Cell Signaling, #9145S)<br>2. Anti-STAT3-P-Y705 antibodies (Cell Signaling, #4904S)

3. Anti-JAK2 (Cell Signaling, #320L)
4. Anti-COL4A2 (Abcam, #125208)

Immunofluorescence:
1. JAK2 (Cell signaling, #3230)

Validation

Western Blots:
1. Anti-STAT3 (Cell Signaling, #9145S); on labome (validated antibody repository), there are N=440 citations. From the manufacturers website, this antibody has been validated by immunoblot using Jurkat and HeLa cells, as well as by using different methodologies in various cell types (immunohsitochemical analysis, confocal cytochemistry, flow cytometry, and chromatin immunoprecipitation)
2. Anti-STAT3-P-Y705 antibodies (Cell Signaling, #4904S); labome (validated antibody repository), there are N=132 citations. From the manufacturers website, experiments using siRNA in HeLa cells were used to validate the quality of the antibody.
3. Anti-JAK2 (Cell Signaling, #3230L); labome (validated antibody repository), there are N=78 citations. From the manufacturers website, experiments using siRNA in K-562 cells were used to validate the quality of the antibody.
4. Anti-COL4A2 (Abcam, #125208); this antibody was validated using RNASEQ data from the same biological sample across multiple different patient samples (Figure 3 in the manuscript). From the manufacturers website, immunoblot experiments using rat kidney lysate and rat lung lysate, as well as immunocytochemical analysis using A431 cells.

Immunofluorescence:
1. JAK2 (Cell signaling, #3230); from the manufacturer website, there are N=305 publications referencing this antibody. From the manufacturers website, immunoblot experiments using siRNA in K-562 cells and immunocytochemcial experiments using lung carcinoma tissue were used to validate the quality of the antibody.

Human research participants

Policy information about studies involving human research participants

Population characteristics

The characteristics of the study population are detailed in Supplementary Table 1 and Extended Data Table 1 of the manuscript.

Recruitment

Hypertrophic cardiomyopathy (HCM) patients were not 'recruited' for this study. Samples from HCM patients included in this study were accessed from a biobank. The biobank includes specimens from patients referred for surgical myectomy at the Tufts Hypertrophic Cardiomyopathy Center. Referral for surgery is based on a clinical indication (i.e., severe heart failure symptoms). Therefore, inclusion of HCM samples in this study was based on sample availability, and to generate a sample pool that reflects the heterogeneous clinical spectrum of this disease. No patient was excluded based on race, sex, age, or other covariates. Self-selection bias is based on the fact that the samples were acquired during a surgical operation and from a single clinical center. For healthy controls, inclusion of these precious samples was based on availability driven mainly by an anatomical mismatch that prevented the use of otherwise normal heart for heart donation.

Ethics oversight

The Institutional Review Boards at Tufts Medical Center, Brigham and Women's Hospital, and the University of Utah.

Note that full information on the approval of the study protocol must also be provided in the manuscript.
